# Body-Force Effects in Homogenised Models of Anisotropic Double Poroelastic Materials

**DOI:** 10.1007/s44007-026-00244-7

**Published:** 2026-09-23

**Authors:** Laura Miller, Raimondo Penta

**Affiliations:** 1 Department of Mathematics and Statistics, University of Strathclyde, 26 Richmond Street, Glasgow, G1 1XH UK; 2 School of Mathematics and Statistics, University of Glasgow, University Place, Glasgow, G12 8QQ UK

**Keywords:** Asymptotic homogenisation, Biot’s poroelasticity, Lorentz force, Multiscale modelling

## Abstract

We develop a rigorous multiscale homogenisation framework for determining the effective behaviour of materials composed of a poroelastic matrix containing embedded poroelastic subphases, each governed by fully anisotropic and heterogeneous Biot equations and subject to body forces. Starting from local balance laws for momentum, Darcy flow, and mass conservation, and assuming scale separation, periodic microstructure, and macroscopic uniformity, we derive a closed system of Biot-type equations at the macroscale. The resulting effective coefficients explicitly capture the influence of microstructural geometry, elastic and hydraulic heterogeneity, and mesoscale forcing. The framework yields well-posed cell problems for the effective elasticity tensor, hydraulic conductivity tensor, and Biot-type coupling coefficients, establishing a unified multiscale theory that extends and subsumes existing single-phase, composite, and double-poroelastic models. As an illustrative application, we specialise the theory to magnetic Lorentz forcing and show that body forces with gradient structure induce additional pressure-like terms in the effective Darcy law, rather than appearing solely as averaged source terms. The proposed framework sets the agenda for modelling complex poroelastic materials with interacting subphases, with applications in biomechanics, engineered composites, and geomechanics.

## Introduction

Purely elastic or purely hydraulic models fail to describe many saturated materials, as they neglect the feedback between deformation of the solid matrix and transport of interstitial fluid. In such settings, stresses, pore pressure, and fluid flux evolve in a coupled manner. This coupled behaviour is captured by the Theory of Poroelasticity, [1–4], which links momentum balance, Darcy flow, and mass conservation within a unified continuum framework. This framework applies to a wide range of physical scenarios where interactions occur between a deformable solid and the pore-scale fluid. Such models are particularly relevant in biological and geomechanical systems, where microscale heterogeneity strongly influences macroscopic response (see [5–12]).

The systems considered here are inherently multiscale, with processes occurring at distinct spatial and temporal scales that interact non-trivially. Multiscale modelling provides a systematic framework for capturing such interactions. It has been employed primarily in materials science to link atomistic or microscale structure to macroscopic mechanical behaviour [13], in geomechanics to couple pore-scale flow with reservoir-scale deformation [14, 15], and in biomechanics to connect cellular or tissue microstructure with organ-level function [16, 17]. Similar multiscale frameworks underpin modern simulations of battery systems [18], climate and weather prediction [19], and turbulent flows [20]. These diverse applications illustrate the broad utility of multiscale approaches and that they are particularly valuable when microscale heterogeneity strongly influences effective material response and cannot be represented accurately within a single-scale description.

**Fig. 1:**
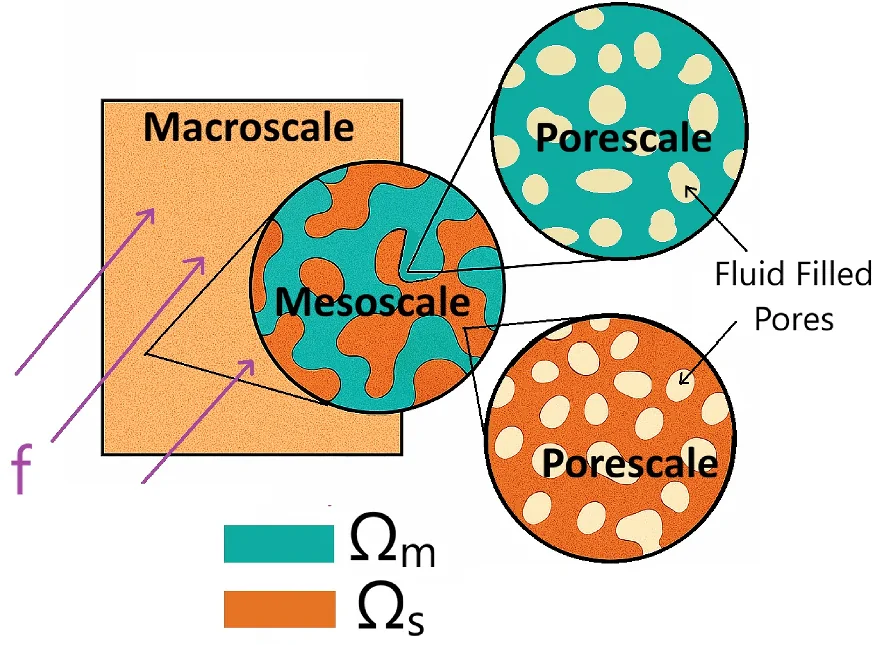
A 2D sketch representing a cross-section of the 3D domain Ω. The matrix $\Omega _m$ is shown in teal and the subphase domain $\Omega _s$ is in orange. There is an interface Γ between the two domains. We also can see that when zooming in on each of the two domains we have two different porous media with a fluid flowing in the pores

In this work, we focus on multiscale deformable porous media with three characteristic length scales. At the smallest scale, fluid–solid interactions occur at the porescale, which is much smaller than the macroscale of the material. Intermediate (meso) scales also arise, such as local heterogeneities in poroelastic properties (see Fig. 1). Our analysis concentrates on the meso- and macroscales, rather than a full three-scale framework ([21] for elastic composites).

We refer to the porescale microstructure because the governing equations employed here arise from upscaling fluid–structure interactions between the solid and fluid phases [22–24]. Specifically, we adopt the most general Biot’s anisotropic, heterogeneous, compressible equations for poroelasticity, rather than embracing simplifications such as the isotropic or incompressible limits. Importantly, in our formulation we also assume that a body force acts on each domain, thereby entering the balance equations at both the mesoscale and macroscale.

The governing equations at the macroscale are obtained by upscaling the local balance equations, incorporating both pore-scale interactions and the imposed body forces. This upscaling can be achieved through homogenisation techniques (see [25] and [26]). Here, we employ the asymptotic homogenisation method [27–30], which exploits the separation between mesoscale and macroscale to decouple spatial variations. In this approach, the relevant fields are expanded as power series in the ratio of the two scales. The resulting balance equations describe the poromechanics of the material at the macroscale, with coefficients encoding microstructural information and the effects of body forces. These coefficients are computed by solving differential problems on the mesoscale.

Asymptotic homogenisation has become a standard tool for deriving effective poroelastic models, beginning with early analyses of wave propagation and effective Biot-type behaviour [22, 31]. Subsequent developments have broadened the framework to incorporate additional physical mechanisms, including appositional growth between fluid and solid phases [32], vascularised poroelastic materials [33], and heterogeneous poroelastic composites [34]. More recently, a substantial body of work has advanced the multiscale theory of poroelastic and multiphase materials through rigorous upscaling from microstructural principles. This includes the derivation of double-poroelastic models [35], homogenised balance laws for nonlinear and electrostrictive composites [36, 37], and micromechanical analyses of effective stiffness in poroelastic media [38]. Further contributions have addressed porosity and diffusion in biological tissues [23], the impact of microstructural alterations such as those induced by myocardial infarction [39], and coupled electro-mechanical homogenisation in cardiac tissue [40]. Collectively, these works demonstrate the versatility of multiscale modelling in capturing the macroscale behaviour of complex poroelastic systems while retaining detailed information about microscale geometry, anisotropy, and multiphysics interactions. However, existing homogenisation frameworks for poroelastic and double-poroelastic media typically neglect phase-dependent body forces or restrict attention to isotropic or incompressible regimes. As a result, the influence of heterogeneous forcing on effective coupling coefficients remains poorly understood.

In this work, we determine the effective behaviour of a material comprising a poroelastic matrix and embedded poroelastic subphases (fibres, inclusions, strata), all interacting under the influence of body forces. Each phase is assumed anisotropic and heterogeneous. Our motivation is to study materials with multiple poroelastic phases interacting at the mesoscale. This structure has been considered previously, e.g. [41], where poroelastic cells were embedded in an extracellular matrix, and equations were derived to study tissue consolidation. Biological tissues, with poroelastic subphases such as cells and collagen fibres [42], provide natural examples.

Here, we adopt the classical two-scale asymptotic homogenisation framework under assumptions of scale separation and periodicity, as is standard in multiscale poromechanics. We generalise [35] and upscale the full interaction between matrix and subphases, each governed by Biot’s anisotropic, heterogeneous, compressible poroelasticity, and each subject to body forces. The mesoscale, where subphases are resolved, is assumed much smaller than the global domain. Upscaling is carried out while enforcing continuity of stresses, displacements, pressures, fluxes, and body force contributions across interfaces. The resulting global model is of Biot-type, with coefficients encoding microstructural properties and body force effects, computed from local differential problems on a finite subset of the domain.

This general formulation is useful since different classes of body force may be transmitted to the macroscale in different ways. Some forcing contributions enter the homogenised equations through averaged source terms, whereas others have additional structure that can modify the effective constitutive relations themselves. To illustrate this distinction, we later specialise the theory to magnetic Lorentz forcing. This is motivated by magnetic-resonance-type settings in biological tissues, where electrically conducting interstitial fluids may experience electromagnetic body forces. In this case, the force may depend on gradients of electromagnetic potentials, and therefore is not captured solely by a simple averaged source term. The Lorentz-force example consequently demonstrates that body-force structure can alter the effective Darcy law itself, producing additional pressure-like contributions at the macroscale.

The paper is organised as follows. In Sect. 2 we consider the quasi-static, multiphase problem consisting of the governing equations for both the poroelastic matrix and the poroelastic subphases with all phases subjected to body forces, and the appropriate interface conditions. The governing equations for the matrix and the subphases are the equations of Biot’s anisotropic, heterogeneous, compressible poroelasticity. In Sect. 3 we introduce the two-scale asymptotic homogenisation method and exploit the scale separation between the inter-subphase distance (the mesoscale) and the overall size of the domain (the macroscale) to derive the macroscale system of PDEs. In Sect. 4 we specialise our force to the magnetic Lorentz force and then in Sect. 5 compare the similarities and differences in the derivations. Section 6 concludes our work by discussing limitations of the model and by providing further perspectives.

## Problem Formulation

We will now introduce our problem. We build on the double-poroelastic framework introduced in [35], extending it to anisotropic, heterogeneous phases subject to phase-dependent body forces.

We consider a set $\Omega \in \mathbb {R}^3$ where Ω is the union of the poroelastic matrix $\Omega _m$, and a collection of disjoint poroelastic subphases where we can write $\bar{\Omega }=\bar{\Omega }_m\cup \bar{\Omega }_s$. Physically, we regard $\Omega _m$ as a connected poroelastic matrix phase and $\Omega _s$ as a collection of embedded poroelastic subphases, which may represent fibres, inclusions, cellular regions, or layered structures depending on the application, see [37] for further geometrical discussion. Both phases are themselves saturated porous materials containing a deformable solid skeleton and an interstitial fluid. The purpose of the present mesoscale model is therefore to describe how two distinct poroelastic materials interact through their common interface and collectively determine the apparent macroscale behaviour of the composite material when subjected to an applied body force. We provide a sketch of a cross-section of the domain Ω in Fig. 1.

To create our model we begin by writing the equations that govern each domain as well as the appropriate interface conditions that will close the problem.

The two phases need balance equations that will govern their mechanical behaviour. These are given by

1a $$\begin{aligned} \nabla \cdot \textsf{T}_m= \textbf{f}_m&\qquad \textrm{in}\quad \Omega _m,\end{aligned}$$

1b $$\begin{aligned} \nabla \cdot \textsf{T}_s=\textbf{f}_s&\qquad \textrm{in}\quad \Omega _s. \end{aligned}$$

The stress tensors $\textsf{T}_m$ and $\textsf{T}_s$ are the stress tensors in each compartment and are those of poroelastic materials. The body forces $\textbf{f}_m$ and $\textbf{f}_s$ here are kept general for the derivation and will be specified later depending on specific applications.

We model each solid phase using Biot’s theory of anisotropic, heterogeneous, compressible poroelasticity [22–24]. Accordingly, the stress tensors ${\textsf{T}}_{m}$ and ${\textsf{T}}_{s}$ appearing in (1a) and (1b) are prescribed by

2a $$\begin{aligned} {\textsf{T}}_{m}=\mathbb {C}_{m}:\zeta (\textbf{u}_{m})-{\boldsymbol{\alpha }}_{m}p_{m}&\qquad \text{ in } \quad \Omega _{m},\end{aligned}$$

2b $$\begin{aligned} {\textsf{T}}_{s}=\mathbb {C}_{s}:\zeta ( \textbf{u}_{s})-{\boldsymbol{\alpha }}_{s}p_{s}&\qquad \text{ in } \quad \Omega _{s}, \end{aligned}$$

where

3 $$\begin{aligned} \zeta (\bullet )=\frac{\nabla (\bullet )+(\nabla (\bullet ))^{\textrm{T}}}{2} \end{aligned}$$

denotes the symmetric part of the gradient operator. We have that $\textbf{u}_{m}$ and $\textbf{u}_{s}$ are the elastic displacements in the matrix and the subphase respectively and $p_{m}$ and $p_{s}$ are the pressures in the matrix and subphase respectively. The $\mathbb {C}_{m}$ and $\mathbb {C}_{s}$ are the effective elasticity tensors that describe the matrix and the subphase. These tensors can be obtained by carrying out a homogenisation process at the finer hierarchical level i.e., at the porescale shown in Fig. 1. These tensors $\mathbb {C}_{m}$ and $\mathbb {C}_{s}$ are the effective elasticity tensors obtained in [22, 23] for a standard poroelastic material. These effective elasticity tensors possess major and minor symmetries as proved in [29]. This means we can write the fourth rank effective elasticity tensors $\mathbb {C}_m$ and $\mathbb {C}_s$ in components as $\mathcal {C}_{pqrs}^{m}$ and $\mathcal {C}_{pqrs}^{s}$, for p,q,r,s=1,2,3. Therefore we have

4a $$\begin{aligned} \mathcal {C}_{pqrs}^{m}=\mathcal {C}_{qprs}^{m}=\mathcal {C}_{pqsr}^{m}=\mathcal {C}_{rspq}^{m},\end{aligned}$$

4b $$\begin{aligned} \mathcal {C}_{pqrs}^{s}=\mathcal {C}_{qprs}^{s}=\mathcal {C}_{pqsr}^{s}=\mathcal {C}_{rspq}^{s}. \end{aligned}$$

The second rank tensors $\boldsymbol{\alpha }_m$ and $\boldsymbol{\alpha }_s$ appearing in (2a) and (2b) are the effective Biot tensors of coefficients in the matrix and subphase respectively. These tensors are obtained from homogenisation at a finer hierarchical scale and quantify the coupling between pore pressure and mechanical deformation. Physically, they describe how changes in fluid pressure contribute to the total stress and how deformation of the solid skeleton influences fluid content. In the classical isotropic Biot theory, these tensors reduce to scalar Biot coefficients multiplied by the identity tensor. The tensorial form adopted here allows this coupling to depend on direction and therefore accommodates anisotropic microstructures and material behaviour.

### Remark 1

For comparison, in the classical isotropic linear poroelastic model the elasticity tensor reduces to the Lamé elasticity tensor and the Biot tensor reduces to $\alpha _{\beta }\textsf{I}$, where β=m,s. The constitutive law then takes the familiar form

$$ \textsf{T}_{\beta } = 2\mu _{\beta }\zeta (\textbf{u}_{\beta }) + \lambda _{\beta }(\nabla \cdot \textbf{u}_{\beta })\textsf{I} - \alpha _{\beta }p_{\beta }\textsf{I}. $$

The present formulation therefore generalises the standard isotropic Biot model to anisotropic and heterogeneous poroelastic phases.

In the subphases and the matrix we also have Darcy’s law governing the fluid flow. That is,

5a $$\begin{aligned} \textbf{w}_{m}=-{\textsf{K}}_{m}(\nabla p_{m}+\textbf{f}_{mf})&\qquad \qquad \text{ in } \quad \Omega _{m},\end{aligned}$$

5b $$\begin{aligned} \textbf{w}_{s}=-{\textsf{K}}_{s}(\nabla p_{s}+\textbf{f}_{sf})&\qquad \qquad \text{ in } \quad \Omega _{s} ,\end{aligned}$$

where ${\textsf{K}}_{m}$ and ${\textsf{K}}_{s}$ are the hydraulic conductivities in the matrix and subphase respectively, and $\textbf{w}_{m}$ and $\textbf{w}_{s}$ are the relative fluid-solid velocities in the matrix and subphase respectively. These are subject to the body forces $\textbf{f}_{mf}$ and $\textbf{f}_{sf}$.

The last governing equation of each compartment is the conservation of mass equation given by

6a $$\begin{aligned} \frac{\dot{p}_{m}}{{M}_{m}}=-{\boldsymbol{\alpha }}_{m}:\zeta ( \dot{\textbf{u}}_{m})-\nabla \cdot \textbf{w}_{m}&\qquad \qquad \text{ in } \quad \Omega _{m},\end{aligned}$$

6b $$\begin{aligned} \frac{\dot{p}_{s}}{{M}_{s}}=-{\boldsymbol{\alpha }}_{s}:\zeta (\dot{\textbf{u}}_{s})-\nabla \cdot \textbf{w}_{s}&\qquad \qquad \text{ in } \quad \Omega _{s}, \end{aligned}$$

in the matrix and subphase. The quantities $M_m$ and $M_s$ are the Biot moduli in the matrix and subphase respectively. These coefficients are obtained from homogenisation at finer hierarchical scales and depend on the local pore geometry, porosity, fluid compressibility, and elastic properties of the solid skeleton. Physically, the Biot modulus characterises the storage capacity of the poroelastic material. Its inverse measures the variation in fluid content induced by a variation in pore pressure under prescribed deformation. Consequently, larger values of $M_m$ and $M_s$ correspond to systems with lower fluid-storage capacity, while smaller values indicate that fluid content changes more readily in response to pressure variations. These have been proved to be positive definite in [29].

The conservation of mass equations describe the balance between fluid storage, deformation-induced changes in pore volume, and fluid transport. The terms involving the Biot tensors account for the change in fluid content caused by local expansion or contraction of the porous skeleton, while the divergence of the Darcy velocity represents the net fluid flux leaving the phase. These equations therefore provide a continuum description of swelling, consolidation, and local reconfiguration of the pore space resulting from coupled fluid-solid interactions.

Finally we need to close the problem by prescribing conditions on the interface Γ. These are continuity of stress, continuity of elastic displacements and the continuity of fluxes and pressures between the subphases and the matrix

7a $$\begin{aligned} \textsf{T}_m \textbf{n}=\textsf{T}_s \textbf{n}&\qquad \textrm{on}\quad \Gamma ,\end{aligned}$$

7b $$\begin{aligned} \textbf{u}_m=\textbf{u}_s&\qquad \textrm{on}\quad \Gamma ,\end{aligned}$$

7c $$\begin{aligned} \textbf{w}_s\cdot \textbf{n}=\textbf{w}_m \cdot \textbf{n}&\qquad \textrm{on}\quad \Gamma ,\end{aligned}$$

7d $$\begin{aligned} p_m=p_s&\qquad \textrm{on}\quad \Gamma , \end{aligned}$$

where $\textbf{n}$ is the normal to the interface Γ pointing into the subphase.

The problem is also to be closed by appropriate boundary conditions on the external boundary $\partial \Omega$. The latter could be, for example, of Dirichlet-Neumann type, as noted in [43]. The conditions on the external boundary typically do not play a role in the derivation of results carried out by formal asymptotic homogenisation.

Now that we have set up the problem we are able to perform a multiscale analysis in order to derive the new model. To do this we will (a) non-dimensionalise the problem we have set up in this section, (b) we will introduce two well-separated length scales, (c) apply the asymptotic homogenisation technique to the non-dimensionalised problem, and (d) derive the novel effective governing equations for the double poroelastic material with applied body force.

## Multiscale Analysis

We begin the multiscale analysis by collecting the governing equations introduced in the previous section. We have

8a $$\begin{aligned} \nabla \cdot \textsf{T}_m=\textbf{f}_m&\qquad \textrm{in}\quad \Omega _m,\end{aligned}$$

8b $$\begin{aligned} \nabla \cdot \textsf{T}_s=\textbf{f}_s&\qquad \textrm{in}\quad \Omega _s,\end{aligned}$$

8c $$\begin{aligned} {\textsf{T}}_{m}=\mathbb {C}_{m}:\zeta (\textbf{u}_{m})-{\boldsymbol{\alpha }}_{m}p_{m}&\qquad \text{ in } \quad \Omega _{m},\end{aligned}$$

8d $$\begin{aligned} {\textsf{T}}_{s}=\mathbb {C}_{s}:\zeta (\textbf{u}_{s})-{\boldsymbol{\alpha }}_{s}p_{s}&\qquad \text{ in } \quad \Omega _{s},\end{aligned}$$

8e $$\begin{aligned} \textbf{w}_{m}=-{\textsf{K}}_{m}(\nabla p_{m}+\textbf{f}_{mf})&\qquad \text{ in } \quad \Omega _{m},\end{aligned}$$

8f $$\begin{aligned} \textbf{w}_{s}=-{\textsf{K}}_{s}(\nabla p_{s}+\textbf{f}_{sf})&\qquad \text{ in } \quad \Omega _{s}\end{aligned}$$

8g $$\begin{aligned} \frac{\dot{p}_{m}}{{M}_{m}}=-{\boldsymbol{\alpha }}_{m}:\zeta (\dot{\textbf{u}}_{m})-\nabla \cdot \textbf{w}_{m}&\qquad \text{ in } \quad \Omega _{m},\end{aligned}$$

8h $$\begin{aligned} \frac{\dot{p}_{s}}{{M}_{s}}=-{\boldsymbol{\alpha }}_{s}:\zeta ( \dot{\textbf{u}}_{s})-\nabla \cdot \textbf{w}_{s}&\qquad \text{ in } \quad \Omega _{s},\end{aligned}$$

8i $$\begin{aligned} \textsf{T}_m \textbf{n}=\textsf{T}_s \textbf{n}&\qquad \textrm{on}\quad \Gamma ,\end{aligned}$$

8j $$\begin{aligned} \textbf{u}_m=\textbf{u}_s&\qquad \textrm{on}\quad \Gamma ,\end{aligned}$$

8k $$\begin{aligned} \textbf{w}_s\cdot \textbf{n}=\textbf{w}_m \cdot \textbf{n}&\qquad \textrm{on}\quad \Gamma ,\end{aligned}$$

8l $$\begin{aligned} p_m=p_s&\qquad \textrm{on}\quad \Gamma , \end{aligned}$$

The problem (8a)–(8l) is closed by prescribing suitable external boundary conditions on $\partial \Omega$. The latter could be, for example, of Dirichlet-Neumann type, as noted in [43]. For the purposes of the formal asymptotic homogenisation carried out here, the precise form of the external boundary conditions does not influence the derivation of the effective equations. The analysis relies on the presence of two well-separated characteristic length scales. We denote the average size of the whole domain by *L* and this is the macroscale. The scale at which we clearly see the subphases resolved from the surrounding matrix is denoted by *d*, the mesoscale.

### The Asymptotic Homogenisation Technique

The homogenisation procedure follows the standard two-scale asymptotic framework developed in [30, 40], here extended to multiphase poroelastic media subject to phase-dependent body forces. We assume that the characteristic length scale of the mesoscale structure, denoted by *d*, is much smaller than the characteristic length scale of the whole domain, denoted by *L*. Thus,

9 $$\begin{aligned} \epsilon =\frac{d}{L}\ll 1. \end{aligned}$$

This separation of scales motivates the introduction of a fast spatial variable

10 $$\begin{aligned} \textbf{y}=\frac{\textbf{x}}{\epsilon }, \end{aligned}$$

where $\textbf{x}$ and $\textbf{y}$ are treated as independent variables representing the macro- and mesoscale coordinates, respectively. The gradient operator is therefore transformed according to

11 $$\begin{aligned} \nabla \rightarrow \nabla _x+\frac{1}{\epsilon }\nabla _y. \end{aligned}$$

Each dependent variable is assumed to admit an asymptotic expansion in integer powers of ϵ, namely

12 $$\begin{aligned} \phi ^{\epsilon }(\textbf{x},\textbf{y},t) = \sum _{l=0}^{\infty } \epsilon ^l \phi ^{(l)}(\textbf{x},\textbf{y},t), \end{aligned}$$

where ϕ denotes any of the fields appearing in the governing equations.

Throughout the derivation, the fields are assumed to be periodic in the mesoscale variable $\textbf{y}$ over a representative cell. This assumption allows the mesoscale problems generated by the asymptotic expansion to be posed on a single periodic cell rather than on the full material microstructure. We also assume macroscopic uniformity of the cell geometry, so that the cell does not vary explicitly with $\textbf{x}$. This permits differentiation under the integral sign, for example

13 $$\begin{aligned} \int _{\Omega } \nabla _x\cdot (\bullet )\,d\textbf{y} = \nabla _x\cdot \int _{\Omega }(\bullet )\,d\textbf{y}, \end{aligned}$$

where (∙) denotes a vector or tensor quantity.

### Multiple Scales Expansion

We now apply the two-scale expansion introduced in Sect. 3.1 to the dimensionless system (8a)–(8l). The full expanded system, obtained after applying $\nabla \mapsto \nabla _x+\epsilon ^{-1}\nabla _y$, is given in Appendix A.1. Here, we record the consequences of the first two orders in ϵ, since these are the terms required to obtain the effective macroscale model.

At leading order, the displacement and pressure fields are independent of the mesoscale variable and are common to both phases. In particular, the $\epsilon ^0$ equations imply

$$ \textbf{u}^{(0)}_m=\textbf{u}^{(0)}_s=\textbf{u}^{(0)}(\textbf{x},t), \qquad p^{(0)}_m=p^{(0)}_s=p^{(0)}(\textbf{x},t). $$

The $\epsilon ^1$ equations determine the first-order corrector fields, which account for mesoscale heterogeneity and phase contrast. These correctors enter the effective constitutive laws and therefore determine the macroscale poroelastic response. We now derive the corresponding cell problems in three steps: first for the elastic displacement correctors and effective stress, then for the pressure correctors and effective Darcy law, and finally for the averaged conservation of mass equation.

### Poroelastic Problem

We begin with the mechanical part of the coupled problem. Combining the leading-order momentum balance with the first-order constitutive relations yields a cell problem for the first-order displacement correctors $\textbf{u}_m^{(1)}$ and $\textbf{u}_s^{(1)}$. These correctors describe how mesoscale elastic heterogeneity and pressure coupling modify the effective stress at the macroscale. Therefore by taking (A4a), (A4b) with (A8c) and (A8d) along with the interface conditions (A4i) and (A8j) we can write

14a $$\begin{aligned} \nabla _y \cdot (\mathbb {C}_{m}\zeta _y(\textbf{u}_{m}^{(1)}))=-\nabla _y\cdot (\mathbb {C}_{m}\zeta _x(\textbf{u}^{(0)}))+\nabla _y\cdot ({p}^{(0)}\boldsymbol{\alpha }_{m})&~~ \text{ in } ~\Omega _{m},\end{aligned}$$

14b $$\begin{aligned} \nabla _y \cdot (\mathbb {C}_{s}\zeta _y(\textbf{u}_{s}^{(1)}))=-\nabla _y\cdot (\mathbb {C}_{s}\zeta _x(\textbf{u}^{(0)}))+\nabla _y\cdot ({p}^{(0)}\boldsymbol{\alpha }_{s})&~~ \text{ in } ~~ \Omega _{s},\end{aligned}$$

14c $$\begin{aligned} (\mathbb {C}_{m}\zeta _y(\textbf{u}_{m}^{(1)})-\mathbb {C}_{s}\zeta _y(\textbf{u}_{s}^{(1)}))\textbf{n}=((\mathbb {C}_{s}-\mathbb {C}_{m})\zeta _x (\textbf{u}^{(0)})-(\boldsymbol{\alpha }_{s}-\boldsymbol{\alpha }_{m}){p}^{(0)})\textbf{n}&~~ \text{ on } ~~\Gamma ,\end{aligned}$$

14d $$\begin{aligned} \textbf{u}_{s}^{(1)}=\textbf{u}_{m}^{(1)}&~~ \text{ on } ~~ \Gamma . \end{aligned}$$

The problem (14a–14d) has a unique solution up to a $\textbf{y}$-constant function. Exploiting the linear structure of the problem, we introduce the following ansatz

15a $$\begin{aligned}&\textbf{u}_{m}^{(1)}=\mathcal {B}_{m}\zeta _x(\textbf{u}^{(0)})+\textbf{b}_{m}{p}^{(0)}+c_1(\textbf{x}),\end{aligned}$$

15b $$\begin{aligned}&\textbf{u}_{s}^{(1)}=\mathcal {B}_{s}\zeta _x(\textbf{u}^{(0)})+\textbf{b}_{s}{p}^{(0)}+c_2(\textbf{x}), \end{aligned}$$

where we have that $c_1(\textbf{x})$ and $c_2(\textbf{x})$ are $\textbf{y}$-constant functions and we have the third order tensors $\mathcal {B}_{m}$ and $\mathcal {B}_{s}$ which satisfy the cell problems given by

16a $$\begin{aligned} \nabla _y \cdot (\mathbb {C}_{m}\zeta _y(\mathcal {B}_{m}))=-\nabla _y\cdot \mathbb {C}_{m}&\qquad \text{ in } \quad \Omega _{m},\end{aligned}$$

16b $$\begin{aligned} \nabla _y \cdot (\mathbb {C}_{s}\zeta _y(\mathcal {B}_{s}))=-\nabla _y\cdot \mathbb {C}_{s}&\qquad \text{ in } \quad \Omega _{s},\end{aligned}$$

16c $$\begin{aligned} (\mathbb {C}_{m}\zeta _y(\mathcal {B}_{m})-\mathbb {C}_{s}\zeta _y(\mathcal {B}_{s}))\textbf{n}=(\mathbb {C}_{s}-\mathbb {C}_{m})\textbf{n}&\qquad \text{ on } \quad \Gamma ,\end{aligned}$$

16d $$\begin{aligned} \mathcal {B}_{m}=\mathcal {B}_{s}&\qquad \text{ on } \quad \Gamma , \end{aligned}$$

as well as the vectors $\textbf{b}_{m}$ and $\textbf{b}_{s}$ which satisfy the problem

17a $$\begin{aligned} \nabla _y \cdot (\mathbb {C}_{m}\zeta _y(\textbf{b}_{m}))=\nabla _y\cdot \boldsymbol{\alpha }_{m}&\qquad \text{ in } \quad \Omega _{m},\end{aligned}$$

17b $$\begin{aligned} \nabla _y \cdot (\mathbb {C}_{s}\zeta _y(\textbf{b}_{s}))=\nabla _y\cdot \boldsymbol{\alpha }_{s}&\qquad \text{ in } \quad \Omega _{s},\end{aligned}$$

17c $$\begin{aligned} (\mathbb {C}_{m}\zeta _y(\textbf{b}_{m})-\mathbb {C}_{s}\zeta _y(\textbf{b}_{s}))\textbf{n}=-(\boldsymbol{\alpha }_{m}-\boldsymbol{\alpha }_{s})\textbf{n}&\qquad \text{ on } \quad \Gamma ,\end{aligned}$$

17d $$\begin{aligned} \textbf{b}_{m}=\textbf{b}_{s}&\qquad \text{ on } \quad \Gamma . \end{aligned}$$

The problems (16a–16d) and (17a–17d) are further supplemented with periodic conditions on $\partial \Omega \setminus \Gamma$. Uniqueness is enforced by prescribing zero average of the sum of auxiliary fields $\mathcal {B}_{m}$, $\mathcal {B}_{s}$, $\textbf{b}_{m}$ and $\textbf{b}_{s}$, over their respective subdomains. That is

18 $$\begin{aligned} \langle \mathcal {B}_m\rangle _m+\langle \mathcal {B}_s\rangle _s=0, \quad \langle \textbf{b}_m\rangle _m+\langle \textbf{b}_s\rangle _s=0. \end{aligned}$$

Now that we have expressions for the leading order elastic displacements we can write the leading order effective stress tensors in the matrix and subphase respectively as

19 $$\begin{aligned} \textsf{T}_{m}^{(0)}&=\mathbb {C}_{m}\zeta _y(\mathcal {B}_{m}\zeta _x(\textbf{u}^{(0)})+\textbf{b}_{m}{p}^{(0)})+\mathbb {C}_{m}\zeta _x(\textbf{u}^{(0)})-\boldsymbol{\alpha }_{m}p^{(0)} \nonumber \\&=(\mathbb {C}_{m}\mathbb {L}_{m}+\mathbb {C}_{m})\zeta _x(\textbf{u}^{(0)})+(\mathbb {C}_{m}{\boldsymbol{\tau }}_{m}-\boldsymbol{\alpha }_{m}){p}^{(0)}, \end{aligned}$$

where we have the auxiliary tensors

20 $$\begin{aligned} \mathbb {L}_{m}=\zeta _y(\mathcal {B}_{m}) \qquad \text{ and } \qquad {\boldsymbol{\tau }}_{m}=\zeta _y(\textbf{b}_{m}), \end{aligned}$$

and

21 $$\begin{aligned} \textsf{T}_{s}^{(0)}&=\mathbb {C}_{s}\zeta _y(\mathcal {B}_{s}\zeta _x(\textbf{u}^{(0)})+\textbf{b}_{s}{p}^{(0)})+\mathbb {C}_{s}\zeta _x(\textbf{u}^{(0)})-\boldsymbol{\alpha }_{s}p^{(0)} \nonumber \\&=(\mathbb {C}_{s}\mathbb {L}_{s}+\mathbb {C}_{s})\zeta _x(\textbf{u}^{(0)})+(\mathbb {C}_{s}{\boldsymbol{\tau }}_{s}-\boldsymbol{\alpha }_{s}){p}^{(0)}, \end{aligned}$$

where we have used the notation

22 $$\begin{aligned} \mathbb {L}_{s}=\zeta _y(\mathcal {B}_{s}) \qquad \text{ and } \qquad {\boldsymbol{\tau }}_{s}=\zeta _y(\textbf{b}_{s}). \end{aligned}$$

The fourth-rank tensors $\mathbb {L}_m$ and $\mathbb {L}_s$ represent mesoscale strain localisation induced by elastic heterogeneity and phase contrast, analogous to strain concentration tensors in classical composite theory. The second rank tensors $\boldsymbol{\tau }_m$ and $\boldsymbol{\tau }_s$ quantify the additional mesoscale deformation induced by pressure loading, beyond that predicted by classical Biot coupling.

We require a balance equation for the effective stresses that takes into consideration both the subphases and the matrix. Integrating the $\epsilon ^1$ momentum balance (A8a) and (A8b) over the matrix and subphase, using periodicity on the external cell boundary and traction continuity on Γ, gives

$$ \nabla _\textbf{x}\cdot \left( \langle {\mathsf T}^{(0)}_m\rangle _m+ \langle {\mathsf T}^{(0)}_s\rangle _s \right) = \langle \textbf{f}^{(0)}_m\rangle _m+ \langle \textbf{f}^{(0)}_s\rangle _s. $$

The detailed cancellation of the cell-boundary and interface terms is reported in Appendix A.1. Thus,

$$ \nabla _\textbf{x}\cdot {\mathsf T}_\textrm{eff} = \langle \textbf{f}^{(0)}_m\rangle _m+ \langle \textbf{f}^{(0)}_s\rangle _s, $$

where

23 $$\begin{aligned} \textsf{T}_{\textrm{eff}}&=\langle \textsf{T}_m^{(0)}\rangle _m+\langle \textsf{T}_s^{(0)}\rangle _s\nonumber \\&=(\langle \mathbb {C}_{m}\mathbb {L}_{m}+\mathbb {C}_{m}\rangle _m+\langle \mathbb {C}_{s}\mathbb {L}_{s}+\mathbb {C}_{s}\rangle _s)\zeta _x(\textbf{u}^{(0)})+(\langle \mathbb {C}_{m}{\boldsymbol{\tau }}_{m}-\boldsymbol{\alpha }_{m}\rangle _m\nonumber \\&\quad +\langle \mathbb {C}_{s}{\boldsymbol{\tau }}_{s}-\boldsymbol{\alpha }_{s}\rangle _s){p}^{(0)} \end{aligned}$$

At this stage, we have derived the constitutive law and the balance equation for the solid portion of our material. We have determined the leading order displacement correctors in both the matrix and subphases ($\mathbb {L}_m$, $\mathbb {L}_s$, $\boldsymbol{\tau }_m$ and $\boldsymbol{\tau }_s$) and identified the associated cell problems defining the elastic and pressure coupling tensors. These correctors encode the influence of mesoscale heterogeneity and phase contrast on the effective stress–strain and stress–pressure relations.

### The Darcy Flow Problem

We can use the balance equations (A4g) and (A4h) with the interface conditions (A4k) and (A8l) to write the following problem for the first order pressures in each phase $p_{m}^{(1)}$ and $p_{s}^{(1)}$

24a $$\begin{aligned} \nabla _y \cdot \textbf{w}_{m}^{(0)}=0&\quad \text{ in } \quad \Omega _{m},\end{aligned}$$

24b $$\begin{aligned} \nabla _y \cdot \textbf{w}_{s}^{(0)}=0&\quad \text{ in } \quad \Omega _{s},\end{aligned}$$

24c $$\begin{aligned} p_{m}^{(1)}=p_{s}^{(1)}&\quad \text{ on }\quad \Gamma ,\end{aligned}$$

24d $$\begin{aligned} \textbf{w}_{m}^{(0)}\cdot \textbf{n}=\textbf{w}_{s}^{(0)}\cdot \textbf{n}&\quad \text{ on }\quad \Gamma . \end{aligned}$$

Using the expressions (A8e) and (A8f) that we have for $\textbf{w}_{m}^{(0)}$ and $\textbf{w}_{s}^{(0)}$ we rewrite the problem (24a–24d) in terms of the pressures

25a $$\begin{aligned} \nabla _y \cdot ({\textsf{K}}_{m}\nabla _y p_{m}^{(1)})=-\nabla _y\cdot ({\textsf{K}}_{m}\nabla _x{p}^{(0)})+\nabla _y\cdot (\textsf{K}_m\textbf{f}_{mf}^{(0)})&~~ \text{ in } ~~\Omega _{m},\end{aligned}$$

25b $$\begin{aligned} \nabla _y \cdot ({\textsf{K}}_{s}\nabla _yp_{s}^{(1)})=-\nabla _y\cdot ({\textsf{K}}_{s}\nabla _x{p}^{(0)})+\nabla _y\cdot (\textsf{K}_s\textbf{f}_{sf}^{(0)})&~~ \text{ in }~~\Omega _{s},\end{aligned}$$

25c $$\begin{aligned} p_{m}^{(1)}=p_{s}^{(1)}&~~ \text{ on }~~\Gamma ,\end{aligned}$$

25d $$\begin{aligned} ({\textsf{K}}_{m}\nabla _y p_{m}^{(1)}-{\textsf{K}}_{s}\nabla _y p_{s}^{(1)})\cdot \textbf{n}=\big (({\textsf{K}}_{s}-{\textsf{K}}_{m})\nabla _x{p}^{(0)}+(\textsf{K}_m\textbf{f}_{mf}^{(0)}-\textsf{K}_s\textbf{f}_{sf}^{(0)})\big )\cdot \textbf{n}&~~ \text{ on }~~\Gamma . \end{aligned}$$

This problem then admits a unique solution up to a $\textbf{y}$-constant function (see [44, 45]). By exploiting the linearity we are able to propose the following ansatz

26a $$\begin{aligned}&p_{m}^{(1)}=\textbf{P}_{m}\cdot \nabla _x{p}^{(0)}+s_m(x,t)+c_3(\textbf{x}),\end{aligned}$$

26b $$\begin{aligned}&p_{s}^{(1)}=\textbf{P}_{s}\cdot \nabla _x{p}^{(0)}+s_s(x,t)+c_4(\textbf{x}), \end{aligned}$$

where we have that $c_3(\textbf{x})$ and $c_4(\textbf{x})$ are $\textbf{y}$-constant functions and the vectors $\textbf{P}_{m}$ and $\textbf{P}_{s}$ and the scalar functions $s_m(x,t)$ and $s_s(x,t)$ satisfy the cell problems given by

27a $$\begin{aligned} \nabla _y\cdot ((\nabla _y\textbf{P}_{m}){\textsf{K}}_{m}^{\textrm{T}})=-\nabla _y\cdot {\textsf{K}}_{m}^{\textrm{T}}&\qquad \text{ in } \quad \Omega _{m},\end{aligned}$$

27b $$\begin{aligned} \nabla _y\cdot ((\nabla _y\textbf{P}_{s}){\textsf{K}}_{s}^{\textrm{T}})=-\nabla _y\cdot {\textsf{K}}_{s}^{\textrm{T}}&\qquad \text{ in } \quad \Omega _{s},\end{aligned}$$

27c $$\begin{aligned} \textbf{P}_{m}=\textbf{P}_{s}&\qquad \text{ on }\quad \Gamma ,\end{aligned}$$

27d $$\begin{aligned} ((\nabla _y\textbf{P}_{m}){\textsf{K}}_{m}^{\textrm{T}}-(\nabla _y\textbf{P}_{s}){\textsf{K}}_{s}^{\textrm{T}})\textbf{n}=({\textsf{K}}_{s}-{\textsf{K}}_{m})^{\textrm{T}}  \textbf{n}&\qquad \text{ on }\quad \Gamma . \end{aligned}$$

and

28a $$\begin{aligned} \nabla _y\cdot ({\textsf{K}}_{m}(\nabla _ys_m))=\nabla _y\cdot ({\textsf{K}}_{m}\textbf{f}_{mf}^{(0)})&\qquad \text{ in } \quad \Omega _{m},\end{aligned}$$

28b $$\begin{aligned} \nabla _y\cdot ({\textsf{K}}_{s}(\nabla _ys_s))=\nabla _y\cdot ({\textsf{K}}_{s}\textbf{f}_{sf}^{(0)})&\qquad \text{ in } \quad \Omega _{s},\end{aligned}$$

28c $$\begin{aligned} {s}_{m}={s}_{s}&\qquad \text{ on }\quad \Gamma ,\end{aligned}$$

28d $$\begin{aligned} (\textsf{K}_{m}(\nabla _y{s}_{m})-\textsf{K}_{s}(\nabla _y{s}_{s}))\cdot \textbf{n}=({\textsf{K}}_{m}\textbf{f}_{mf}^{(0)}-{\textsf{K}}_{s}\textbf{f}_{sf}^{(0)})\cdot \textbf{n}&\qquad \text{ on }\quad \Gamma . \end{aligned}$$

The cell problem is supplemented by periodic conditions on the boundary $\partial \Omega \setminus \Gamma$ and for the sake of uniqueness of solution we place a further condition on $\textbf{P}_{m}$ and $\textbf{P}_{s}$. We impose a zero-average condition on the sum of the auxiliary fields over the two subdomains, that is

29 $$\begin{aligned} \langle \textbf{P}_{m}\rangle _m+\langle \textbf{P}_{s}\rangle _{s}=0. \end{aligned}$$

We now wish to find the expressions for the leading order Darcy’s law in the subphases and matrix respectively. We can use the expressions (26a) and (26b) for the first order pressures $p_{m}^{(1)}$ and $p_{s}^{(1)}$ in (A8e) and (A8f) and by applying the integral average we obtain

30 $$\begin{aligned} \langle \textbf{w}_{m}^{(0)}\rangle _{m}&=-\langle {\textsf{K}}_{m}(\nabla _y\textbf{P}_{m})^{\textrm{T}}\rangle _{m}\nabla _x{p}^{(0)}-\langle {\textsf{K}}_{m}\rangle _{m}\nabla _x{p}^{(0)}-\langle {\textsf{K}}_{m}\nabla _y{s}_{m}\rangle _m+\langle \textsf{K}_{m}\textbf{f}_{mf}^{(0)}\rangle _m\nonumber \\&=-\langle {\textsf{K}}_{m}{\textsf{R}}_{m}+{\textsf{K}}_{m}\rangle _{m}\nabla _x {p}^{(0)}-\langle {\textsf{K}}_{m}  \textbf{s}_{m}\rangle _m+\langle \textsf{K}_{m}\textbf{f}_{mf}^{(0)}\rangle _m, \end{aligned}$$

in the matrix where we have used the notation

31 $$\begin{aligned} {\textsf{R}}_{m}=(\nabla _y\textbf{P}_{m})^{\textrm{T}} \quad \text{ and } \quad \textbf{s}_m=\nabla _ys_m \end{aligned}$$

and we obtain

32 $$\begin{aligned} \langle \textbf{w}_{s}^{(0)}\rangle _{s}&=-\langle {\textsf{K}}_{s}(\nabla _y\textbf{P}_{s})^{\textrm{T}}\rangle _{s}\nabla _x{p}^{(0)}-\langle {\textsf{K}}_{s}\rangle _{s}\nabla _x{p}^{(0)}-\langle {\textsf{K}}_{s}\nabla _y{s}_{s}\rangle _s+\langle \textsf{K}_{s}\textbf{f}_{sf}^{(0)}\rangle _s\nonumber \\&=-\langle {\textsf{K}}_{s}{\textsf{R}}_{s}+{\textsf{K}}_{s}\rangle _{s}\nabla _x {p}^{(0)}-\langle {\textsf{K}}_{s}  \textbf{s}_{s}\rangle _s+\langle \textsf{K}_{s}\textbf{f}_{sf}^{(0)}\rangle _s, \end{aligned}$$

in the subphase where we have used the notation

33 $$\begin{aligned} {\textsf{R}}_{s}=(\nabla _y\textbf{P}_{s})^{\textrm{T}} \quad \text{ and } \quad \textbf{s}_s=\nabla _ys_s \end{aligned}$$

These expressions are now combined to derive the effective Darcy law governing the macroscopic flow. We have

34 $$\begin{aligned} \textbf{w}_{\textrm{eff}}:&=\langle \textbf{w}_{m}^{(0)}\rangle _m+\langle \textbf{w}_{s}^{(0)}\rangle _s\nonumber \\  &=-\big (\langle {\textsf{K}}_{m}{\textsf{R}}_{m}+{\textsf{K}}_{m}\rangle _m+\langle {\textsf{K}}_{s}{\textsf{R}}_{s}+{\textsf{K}}_{s}\rangle _s\big )\nabla _x {p}^{(0)}-\langle {\textsf{K}}_{m}  \textbf{s}_{m}\rangle _m+\langle \textsf{K}_{m}\textbf{f}_{mf}^{(0)}\rangle _m\nonumber \\&\quad -\langle {\textsf{K}}_{s}  \textbf{s}_{s}\rangle _s+\langle \textsf{K}_{s}\textbf{f}_{sf}^{(0)}\rangle _s, \end{aligned}$$

where we can define an effective hydraulic conductivity tensor as

35 $$\begin{aligned} \textsf{W}=\langle {\textsf{K}}_{m}{\textsf{R}}_{m}+{\textsf{K}}_{m}\rangle _m+\langle {\textsf{K}}_{s}{\textsf{R}}_{s}+{\textsf{K}}_{s}\rangle _s \end{aligned}$$

and this means that Darcy’s law can be rewritten as

36 $$\begin{aligned} \textbf{w}_{\textrm{eff}}=-{\textsf{W}}\nabla _x {p}^{(0)}-\langle {\textsf{K}}_{m}  \textbf{s}_{m}\rangle _m+\langle \textsf{K}_{m}\textbf{f}_{mf}^{(0)}\rangle _m\ -\langle {\textsf{K}}_{s}  \textbf{s}_{s}\rangle _s+\langle \textsf{K}_{s}\textbf{f}_{sf}^{(0)}\rangle _s. \end{aligned}$$

The resulting expressions define an effective Darcy law in which the hydraulic conductivity tensor incorporates both phase-wise conductivities and mesoscale correctors accounting for heterogeneity and body-force effects.

### Conservation of Mass

The final equation required to close the macroscale system is the averaged conservation of mass equation. Integrating the $\epsilon ^1$ mass balance equations over the matrix and subphase, and using flux continuity across Γ, gives an effective mass balance involving the macroscale pressure, the effective relative fluid–solid velocity, and the first-order displacement correctors. The intermediate averaging steps are given in Appendix A.2.

Substituting the first-order displacement correctors (15a)–(15b) into this averaged balance yields

37 $$\begin{aligned} \dot{p}^{(0)}&= -\bar{\mathcal {M}} \bigg ( \nabla _x\cdot \textbf{w}_{\textrm{eff}} + \bar{\boldsymbol{\alpha }}:\zeta _x(\dot{\textbf{u}}^{(0)}) \bigg ), \end{aligned}$$

where

38 $$\begin{aligned} \bar{\mathcal {M}} {:}{=} \frac{\langle {M}_{m}\rangle _{m}\langle {M}_{s}\rangle _{s}}{\langle {M}_{m}\rangle _{m}+\langle {M}_{s}\rangle _{s} +\langle {M}_{m}\rangle _{m}\langle {M}_{s}\rangle _{s} \left( \langle \boldsymbol{\alpha }_{m}:{\boldsymbol{\tau }}_{m}\rangle _{m} + \langle \boldsymbol{\alpha }_{s}:{\boldsymbol{\tau }}_{s}\rangle _{s} \right) }, \end{aligned}$$

and

39 $$\begin{aligned} \bar{\boldsymbol{\alpha }} {:}{=} \left\langle \boldsymbol{\alpha }_{m} + \boldsymbol{\alpha }_{s} + \mathbb {L}_{m}^{\textrm{T}}:\boldsymbol{\alpha }_{m} + \mathbb {L}_{s}^{\textrm{T}}:\boldsymbol{\alpha }_{s} \right\rangle _{\scriptscriptstyle \Omega }. \end{aligned}$$

The scalar coefficient $\bar{\mathcal {M}}$ plays the role of an effective Biot modulus for the double poroelastic system, while $\bar{\boldsymbol{\alpha }}$ is the corresponding effective Biot tensor. The latter contains the phase-wise Biot tensors together with corrector terms arising from mesoscale compressibility contrasts and displacement–pressure coupling across the matrix–subphase interface.

We have now derived all the equations required to be able to state our macroscale model.

### Macroscale Model

By assembling the results derived above, we obtain a closed system of macroscale equations describing the effective poroelastic response of this double poroelastic material subject to body forces. The system consists of a balance of linear momentum, a modified Darcy law, and a conservation of mass equation, all expressed in terms of the leading order elastic displacement $\textbf{u}^{(0)}$, the leading order body forces, the relative fluid-solid velocity $\textbf{w}_{\textrm{eff}}$ and the pressure $p^{(0)}$. The model is given by

40a $$\begin{aligned}&\nabla _\textbf{x}\cdot \textsf{T}_{\textrm{eff}}=\langle \textbf{f}_m^{(0)}\rangle _m+\langle \textbf{f}_s^{(0)}\rangle _s,\end{aligned}$$

40b $$\begin{aligned}&\textsf{T}_{\textrm{eff}}=(\langle \mathbb {C}_{m}\mathbb {L}_{m}+\mathbb {C}_{m}\rangle _m+\langle \mathbb {C}_{s}\mathbb {L}_{s}+\mathbb {C}_{s}\rangle _s)\zeta _x(\textbf{u}^{(0)})+(\langle \mathbb {C}_{m}{\boldsymbol{\tau }}_{m}-\boldsymbol{\alpha }_{m}\rangle _m \nonumber \\&\qquad \quad +\langle \mathbb {C}_{s}{\boldsymbol{\tau }}_{s}-\boldsymbol{\alpha }_{s}\rangle _s){p}^{(0)}\end{aligned}$$

40c $$\begin{aligned}&\dot{p}^{(0)}=-\bar{\mathcal {M}}\bigg (\nabla _x\cdot \textbf{w}_{\textrm{eff}}+\langle \boldsymbol{\alpha }_{m}+\boldsymbol{\alpha }_{s} +\mathbb {L}_{m}^{\textrm{T}}:\boldsymbol{\alpha }_{m}+\mathbb {L}_{s}^{\textrm{T}}: \boldsymbol{\alpha }_{s}\rangle _{\scriptscriptstyle \Omega }:\zeta _x (\dot{\textbf{u}}^{(0)})\bigg ),\end{aligned}$$

40d $$\begin{aligned}&\textbf{w}_{\textrm{eff}}=-{\textsf{W}}\nabla _x {p}^{(0)}-\langle {\textsf{K}}_{m}  \textbf{s}_{m}\rangle _m+\langle \textsf{K}_{m}\textbf{f}_{mf}^{(0)}\rangle _m\ -\langle {\textsf{K}}_{s}  \textbf{s}_{s}\rangle _s+\langle \textsf{K}_{s}\textbf{f}_{sf}^{(0)}\rangle _s \end{aligned}$$

We have the macroscale balance equation (40a) for the effective stress tensor (40b) subject to the leading order body forces. The effective stress comprises the coefficient tensors $\mathbb {C}_m+\mathbb {C}_m\mathbb {L}_m$ and $\mathbb {C}_s+\mathbb {C}_s\mathbb {L}_s$ which are found by solving (16a)–(16d) and account for differences in elastic properties at different points in the microstructure. The terms $\mathbb {L}_m$ and $\mathbb {L}_s$ can be thought of as corrector terms to the classical averaging of the elastic properties. The effective stress also contains a term relating to the fluid pressure where the coefficient of this term is to be found by solving (17a)–(17d). This coefficient encodes differences in the compressibility at different points in the microstructure. The problems to be solved for the coefficients in the effective stress are similar to those found for elastic composites in [46, 47], poroelastic composites in [34, 38, 39] and double poroelastic materials [35].

We then have (40c) which is the macroscale conservation of mass equation. This equation comprises the divergence of the relative fluid-solid velocity $\textbf{w}_{\textrm{eff}}$ and the Biot’s tensor of coefficients $\bar{\boldsymbol{\alpha }}$ applied to the leading order strains. The Biot’s tensor that we obtain here (39) comprises the Biot’s tensors from the subphase and the matrix as well as the two additional corrector contributions arising due to the fact that we are accounting for changing compressibility at different points in the microstructure.

And finally we have Darcy’s law (40d) with the modified hydraulic conductivity tensor $\textsf{W}$ (35). This tensor consists of the hydraulic conductivities $\textsf{K}_m$ and $\textsf{K}_s$ from the subphase and matrix as well as two additional terms. The extra terms $\textsf{K}_m\textsf{R}_m$ and $\textsf{K}_s\textsf{R}_s$ account for the differences in the hydraulic conductivities at different points in the microstructure. The hydraulic conductivity tensor $\textsf{W}$ can be found by solving cell problem (27a)–(27d). Darcy’s law also contains the additional leading order forcing contributions and phase-averaged sources to be computed by solving cell problem (28a)–(28d).

Although the derivation above is written in the most general anisotropic form, the effective tensors simplify considerably when the periodic cell possesses additional material or geometrical symmetries. For example, isotropic, transversely isotropic, orthotropic, or layered microstructures reduce the number of independent entries of the effective elasticity tensor, hydraulic conductivity tensor, and Biot-type coupling tensors. In such cases fewer independent cell problems are required. Thus, the fully anisotropic formulation presented here should be viewed as the general framework from which practically simpler models can be obtained by imposing the relevant symmetry class.

## Specialising to the Application of Magnetic Lorentz Force

In this section, we specialise the general body-force framework derived above to the case of magnetic Lorentz forcing. This example is chosen because it represents a structured body force: rather than entering the governing equations as a prescribed source alone, it depends on gradients of electromagnetic potentials and is therefore coupled to the transport structure of the problem. Such forces are relevant in magnetic-resonance-type settings involving saturated biological tissues or porous materials with electrically conducting interstitial fluids.

The purpose of this section is not to develop a full electromagnetic model, but to demonstrate how the mathematical structure of a particular body force is transmitted through the homogenisation procedure. In particular, the Lorentz-force example shows that body forces with gradient structure can generate additional pressure-like contributions in the effective Darcy law, rather than appearing only as averaged forcing terms in the macroscale equations.

We define the magnetic Lorentz forcing in the matrix and subphase by

41 $$\begin{aligned} \textbf{f}_{mf}=\textbf{j}_m\times \textbf{B}, \qquad \textbf{j}_m=-{\mathsf G}_m\nabla \phi _m, \end{aligned}$$

and

42 $$\begin{aligned} \textbf{f}_{sf}=\textbf{j}_s\times \textbf{B}, \qquad \textbf{j}_s=-{\mathsf G}_s\nabla \phi _s. \end{aligned}$$

Here, $\textbf{j}_\alpha$ denotes the current density, ${\mathsf G}_\alpha$ is the electrical conductance tensor, $\phi _\alpha$ is the electric potential, $\textbf{B}$ is the imposed magnetic field, and α=m,s denotes the matrix and subphase respectively.

We present below the key changes induced by Lorentz forcing. The full order-by-order expansion, the longer auxiliary cell problems, and the intermediate averaging steps are provided in Appendix B.

### Lorentz-Modified Darcy’s Law

We first examine the phase-wise Darcy laws, since this is where the Lorentz force enters the coupled system most directly. Substituting (41) and (42) into the Darcy laws gives

43 $$\begin{aligned} \textbf{w}_m&= -\mathsf K_m\nabla p_m + \mathsf K_m(\mathsf G_m\nabla \phi _m\times \textbf{B}), \end{aligned}$$

44 $$\begin{aligned} \textbf{w}_s&= -\mathsf K_s\nabla p_s + \mathsf K_s(\mathsf G_s\nabla \phi _s\times \textbf{B}). \end{aligned}$$

The important distinction from the generic body-force case is that the Lorentz terms contain gradients of electromagnetic potentials. As a result, the leading-order pressure is no longer, in general, represented by a single phase-independent quantity. Instead, the order $\epsilon ^0$ problem gives

45a $$\begin{aligned} \nabla _y \cdot (\textsf{K}_m \nabla _y p_m^{(0)})-\nabla _y \cdot \left( \textsf{K}_{m}(\textsf{G}_{m} \nabla _y\phi _{m}^{(0)} \times \textbf{B}^{(0)})\right) =0&~~ \text{ in } \quad \Omega _m,\end{aligned}$$

45b $$\begin{aligned} \nabla _y \cdot (\textsf{K}_s \nabla _y p_s^{(0)})-\nabla _y \cdot \left( \textsf{K}_s(\textsf{G}_s \nabla _y\phi _s^{(0)} \times \textbf{B}^{(0)})\right) =0&~~ \text{ in } \quad \Omega _s,\end{aligned}$$

45c $$\begin{aligned} \left( \textsf{K}_m \nabla _y p_m^{(0)} -\textsf{K}_{m}(\textsf{G}_{m} \nabla _y\phi _{m}^{(0)} \times \textbf{B}^{(0)})\right) \cdot \textbf{n} = \left( \textsf{K}_s \nabla _y p_s^{(0)} -\textsf{K}_s(\textsf{G}_s \nabla _y\phi _s^{(0)} \times \textbf{B}^{(0)})\right) \cdot \textbf{n}&~~\text{ on } \quad \Gamma ,\end{aligned}$$

45d $$\begin{aligned} p_m^{(0)}=p_s^{(0)}&~~~\text{ on } \quad \Gamma . \end{aligned}$$

Thus, by linearity, we write

46 $$\begin{aligned} p_m^{(0)}&=\hat{p}_m+\bar{p}(\textbf{x},t),\end{aligned}$$

47 $$\begin{aligned} p_s^{(0)}&=\hat{p}_s+\bar{p}(\textbf{x},t), \end{aligned}$$

where $\hat{p}_m$ and $\hat{p}_s$ are Lorentz-induced mesoscale pressure correctors and $\bar{p}(\textbf{x},t)$ is independent of $\textbf{y}$. The pressure correctors satisfy

48a $$\begin{aligned} \nabla _y \cdot (\textsf{K}_m \nabla _y \hat{p}_m)- \nabla _y \cdot \left( \textsf{K}_{m}(\textsf{G}_{m} \nabla _y\phi _{m}^{(0)} \times \textbf{B}^{(0)})\right) =0&~~\text{ in } \quad \Omega _m,\end{aligned}$$

48b $$\begin{aligned} \nabla _y \cdot (\textsf{K}_s \nabla _y \hat{p}_s) -\nabla _y \cdot \left( \textsf{K}_s(\textsf{G}_s \nabla _y\phi _s^{(0)} \times \textbf{B}^{(0)})\right) =0&~~\text{ in } \quad \Omega _s,\end{aligned}$$

48c $$\begin{aligned} \left( \textsf{K}_m \nabla _y \hat{p}_m - \textsf{K}_{m}(\textsf{G}_{m} \nabla _y\phi _{m}^{(0)} \times \textbf{B}^{(0)})\right) \cdot \textbf{n} = \left( \textsf{K}_s \nabla _y \hat{p}_s - \textsf{K}_s(\textsf{G}_s \nabla _y\phi _s^{(0)} \times \textbf{B}^{(0)})\right) \cdot \textbf{n}&~~\text{ on } \quad \Gamma ,\end{aligned}$$

48d $$\begin{aligned} \hat{p}_m=\hat{p}_s&~~\text{ on } \quad \Gamma . \end{aligned}$$

These equations are supplemented with periodic boundary conditions on $\partial \Omega \setminus \Gamma$ and a zero-average condition to ensure uniqueness.

Proceeding to the order $\epsilon ^1$ problem, we use the ansatz

49 $$\begin{aligned} p_m^{(1)}&=\textbf{d}_m\cdot \nabla _x\bar{p}+t_m+c(\textbf{x}),\end{aligned}$$

50 $$\begin{aligned} p_s^{(1)}&=\textbf{d}_s\cdot \nabla _x\bar{p}+t_s+c(\textbf{x}), \end{aligned}$$

where $\textbf{d}_m,\textbf{d}_s$ are hydraulic correctors and $t_m,t_s$ collect the remaining Lorentz-induced contributions. Their cell problems are stated in Appendix B.1. Defining

51 $$\begin{aligned} {\mathsf D}_m=(\nabla _y\textbf{d}_m)^{\mathrm T}, \qquad {\mathsf D}_s=(\nabla _y\textbf{d}_s)^{\mathrm T}, \qquad \textbf{t}_m=\nabla _y t_m, \qquad \textbf{t}_s=\nabla _y t_s, \end{aligned}$$

the effective Darcy law is

52 $$\begin{aligned} \textbf{w}_{\textrm{eff}}&=-{\mathsf W}\nabla _x\bar{p} -\langle \mathsf K_m\nabla _x\hat{p}_m\rangle _m -\langle \mathsf K_s\nabla _x\hat{p}_s\rangle _s -\langle \mathsf K_m\textbf{t}_m\rangle _m -\langle \mathsf K_s\textbf{t}_s\rangle _s +\textbf{F}_{L}, \end{aligned}$$

where

53 $$\begin{aligned} \mathsf W = \langle \mathsf K_m\rangle _m+ \langle \mathsf K_s\rangle _s+ \langle \mathsf K_m\mathsf D_m\rangle _m+ \langle \mathsf K_s\mathsf D_s\rangle _s, \end{aligned}$$

and

54 $$\begin{aligned} \textbf{F}_{L}&= \langle \mathsf K_m(\mathsf G_m\nabla _x\phi _m^{(0)}\times \textbf{B}^{(0)})\rangle _m + \langle \mathsf K_s(\mathsf G_s\nabla _x\phi _s^{(0)}\times \textbf{B}^{(0)})\rangle _s \nonumber \\&\quad + \langle \mathsf K_m(\mathsf G_m\nabla _y\phi _m^{(0)}\times \textbf{B}^{(1)})\rangle _m + \langle \mathsf K_m(\mathsf G_m\nabla _y\phi _m^{(1)}\times \textbf{B}^{(0)})\rangle _m \nonumber \\&\quad + \langle \mathsf K_s(\mathsf G_s\nabla _y\phi _s^{(0)}\times \textbf{B}^{(1)})\rangle _s + \langle \mathsf K_s(\mathsf G_s\nabla _y\phi _s^{(1)}\times \textbf{B}^{(0)})\rangle _s. \end{aligned}$$

Equation (52) shows that the Lorentz force does not simply enter as an averaged source term. It generates mesoscale pressure correctors and additional driving terms in the effective pressure–flow relation.

### Lorentz-Modified Poroelastic Problem

We next consider the effect of Lorentz forcing on the mechanical part of the coupled problem. For simplicity, we assume that the Lorentz force acting on the solid components is the same in the matrix and subphase:

55 $$\begin{aligned} \textbf{f}_{m}=\textbf{f}_{s}=-\mathsf G\nabla \phi \times \textbf{B}. \end{aligned}$$

Here, G is the conductance tensor, ϕ is the electric potential and $\textbf{B}$ is the magnetic field. These quantities are, in general, distinct from $\mathsf G_m$, $\mathsf G_s$, $\phi _m$ and $\phi _s$ appearing in (41)–(42).

The Lorentz-modified poroelastic cell problem differs from Sect. 3.3 because the leading-order pressures in the two phases are retained separately. The first-order displacement correctors are therefore written as

56a $$\begin{aligned} \textbf{u}_{m}^{(1)}&=\mathcal {B}_m\zeta _x(\textbf{u}^{(0)})+\textbf{b}_m p_m^{(0)}+\tilde{\textbf{b}}_m p_s^{(0)}+\textbf{e}_m+c_1(\textbf{x}),\end{aligned}$$

56b $$\begin{aligned} \textbf{u}_{s}^{(1)}&=\mathcal {B}_s\zeta _x(\textbf{u}^{(0)})+\textbf{b}_s p_s^{(0)}+\tilde{\textbf{b}}_s p_m^{(0)}+\textbf{e}_s+c_2(\textbf{x}). \end{aligned}$$

The correctors $\mathcal {B}_m,\mathcal {B}_s,\textbf{b}_m,\textbf{b}_s,\tilde{\textbf{b}}_m$ and $\tilde{\textbf{b}}_s$ are defined in Appendix B.2. The genuinely new Lorentz-induced mechanical correctors $\textbf{e}_m$ and $\textbf{e}_s$ solve

57a $$\begin{aligned} \nabla _y\cdot (\mathbb {C}_m\zeta _y(\textbf{e}_m))&=-\mathsf G\nabla _y\phi ^{(0)}\times \textbf{B}  &   \text{ in } \quad \Omega _m,\end{aligned}$$

57b $$\begin{aligned} \nabla _y\cdot (\mathbb {C}_s\zeta _y(\textbf{e}_s))&=-\mathsf G\nabla _y\phi ^{(0)}\times \textbf{B}  &   \text{ in } \quad \Omega _s,\end{aligned}$$

57c $$\begin{aligned} (\mathbb {C}_m\zeta _y(\textbf{e}_m))\textbf{n}&=(\mathbb {C}_s\zeta _y(\textbf{e}_s))\textbf{n}  &   \text{ on } \quad \Gamma ,\end{aligned}$$

57d $$\begin{aligned} \textbf{e}_m&=\textbf{e}_s  &   \text{ on } \quad \Gamma . \end{aligned}$$

These problems are supplemented with periodic boundary conditions on $\partial \Omega \setminus \Gamma$ and zero-average conditions for uniqueness.

Define

58 $$\begin{aligned} \mathbb {L}_m=\zeta _y(\mathcal {B}_m),\qquad \boldsymbol{\tau }_m=\zeta _y(\textbf{b}_m),\qquad \tilde{\boldsymbol{\tau }}_m=\zeta _y(\tilde{\textbf{b}}_m),\qquad \boldsymbol{\theta }_m=\zeta _y(\textbf{e}_m), \end{aligned}$$

and

59 $$\begin{aligned} \mathbb {L}_s=\zeta _y(\mathcal {B}_s),\qquad \boldsymbol{\tau }_s=\zeta _y(\textbf{b}_s),\qquad \tilde{\boldsymbol{\tau }}_s=\zeta _y(\tilde{\textbf{b}}_s),\qquad \boldsymbol{\theta }_s=\zeta _y(\textbf{e}_s). \end{aligned}$$

The leading-order stresses are then

60 $$\begin{aligned} \mathsf T_m^{(0)}&=(\mathbb {C}_m\mathbb {L}_m+\mathbb {C}_m)\zeta _x(\textbf{u}^{(0)}) +(\mathbb {C}_m\boldsymbol{\tau }_m-\boldsymbol{\alpha }_m)p_m^{(0)} +\mathbb {C}_m\tilde{\boldsymbol{\tau }}_m p_s^{(0)} +\mathbb {C}_m\boldsymbol{\theta }_m, \end{aligned}$$

61 $$\begin{aligned} \mathsf T_s^{(0)}&=(\mathbb {C}_s\mathbb {L}_s+\mathbb {C}_s)\zeta _x(\textbf{u}^{(0)}) +(\mathbb {C}_s\boldsymbol{\tau }_s-\boldsymbol{\alpha }_s)p_s^{(0)} +\mathbb {C}_s\tilde{\boldsymbol{\tau }}_s p_m^{(0)} +\mathbb {C}_s\boldsymbol{\theta }_s. \end{aligned}$$

For the solid Lorentz force, the leading-order body force entering the effective momentum balance is

62 $$\begin{aligned} \textbf{f}^{(0)}=-\mathsf G\nabla _y\phi ^{(1)}\times \textbf{B}^{(0)} -\mathsf G\nabla _y\phi ^{(0)}\times \textbf{B}^{(1)} -\mathsf G\nabla _x\phi ^{(0)}\times \textbf{B}^{(0)}. \end{aligned}$$

Hence the Lorentz-modified effective momentum balance is

63 $$\begin{aligned} \nabla _x\cdot \mathsf T_{\textrm{eff}} = \langle \textbf{f}^{(0)}\rangle _m+ \langle \textbf{f}^{(0)}\rangle _s, \end{aligned}$$

where

64 $$\begin{aligned} \mathsf T_{\textrm{eff}}&= \langle \mathsf T_m^{(0)}\rangle _m+\langle \mathsf T_s^{(0)}\rangle _s \nonumber \\&= \left( \langle \mathbb {C}_m\mathbb {L}_m+\mathbb {C}_m\rangle _m +\langle \mathbb {C}_s\mathbb {L}_s+\mathbb {C}_s\rangle _s\right) \zeta _x(\textbf{u}^{(0)}) +\left\langle \mathbb {C}_m\boldsymbol{\tau }_m-\boldsymbol{\alpha }_m\right\rangle _m p_m^{(0)}\nonumber \\&\quad +\left\langle \mathbb {C}_s\boldsymbol{\tau }_s-\boldsymbol{\alpha }_s\right\rangle _s p_s^{(0)} +\left\langle \mathbb {C}_m\tilde{\boldsymbol{\tau }}_m\right\rangle _m p_s^{(0)} +\left\langle \mathbb {C}_s\tilde{\boldsymbol{\tau }}_s\right\rangle _s p_m^{(0)} \nonumber \\&\quad +\left\langle \mathbb {C}_m\boldsymbol{\theta }_m\right\rangle _m +\left\langle \mathbb {C}_s\boldsymbol{\theta }_s\right\rangle _s . \end{aligned}$$

#### Remark 2

We have assumed that the external loads are the same in the solid phase, while they may differ in the fluid phase. In the fluid phase, the role of the external body force is encoded in Darcy’s law, and local conservation of mass enforces continuity of the normal component of the velocity at the interface, as in [48]. For elastic-type problems with vector or scalar potential body forces, the boundary conditions involve continuity of tractions and displacements. In this setting, compatibility of the cell problems requires care when external loads are discontinuous across the interface; alternative formulations, such as decompositions involving inelastic stress contributions [37], can accommodate discontinuous loads by incorporating them into the traction condition.

### Lorentz-Modified Conservation of Mass

We now derive the macroscale conservation of mass equation in the presence of Lorentz forcing, without assuming the existence of a phase-independent leading-order pressure. Accordingly, the leading-order pressures in the matrix and subphase are denoted by $p_m^{(0)}$ and $p_s^{(0)}$, respectively.

Integrating the phase-wise mass balances over the cell, using flux continuity across Γ, and substituting the displacement correctors (56a)–(56b) gives

65 $$\begin{aligned}&\mathcal {A}_m\dot{p}_m^{(0)}+ \mathcal {A}_s\dot{p}_s^{(0)} = -\nabla _x\cdot \textbf{w}_{\textrm{eff}} -\bar{\boldsymbol{\alpha }}:\zeta _x(\dot{\textbf{u}}^{(0)}), \end{aligned}$$

where

66 $$\begin{aligned} \mathcal {A}_m&{:}{=} \langle M_m\rangle _m^{-1} +\langle \boldsymbol{\alpha }_m:\boldsymbol{\tau }_m\rangle _m +\langle \boldsymbol{\alpha }_s:\tilde{\boldsymbol{\tau }}_s\rangle _s, \end{aligned}$$

67 $$\begin{aligned} \mathcal {A}_s&{:}{=} \langle M_s\rangle _s^{-1} +\langle \boldsymbol{\alpha }_s:\boldsymbol{\tau }_s\rangle _s +\langle \boldsymbol{\alpha }_m:\tilde{\boldsymbol{\tau }}_m\rangle _m, \end{aligned}$$

and

68 $$\begin{aligned} \bar{\boldsymbol{\alpha }} {:}{=} \left\langle \boldsymbol{\alpha }_m+ \mathbb {L}_m^{\mathrm T}:\boldsymbol{\alpha }_m \right\rangle _m + \left\langle \boldsymbol{\alpha }_s+ \mathbb {L}_s^{\mathrm T}:\boldsymbol{\alpha }_s \right\rangle _s. \end{aligned}$$

The intermediate averaging steps are given in Appendix B.3. The coefficients $\mathcal {A}_m$ and $\mathcal {A}_s$ are effective storage coefficients. Equivalently, one may define phase-wise effective Biot-type moduli by $\bar{M}_m=\mathcal {A}_m^{-1}$ and $\bar{M}_s=\mathcal {A}_s^{-1}$.

Equation (65) shows that Lorentz effects enter the conservation of mass through the modified effective flux, while the separation of the leading-order pressures produces additional storage and coupling terms.

### The Macroscale Model for a Double Poroelastic Material Subject to the Magnetic Lorentz Force

Collecting the previous results, the Lorentz-modified macroscale model is

69a $$\begin{aligned}&\nabla _x\cdot \mathsf T_{\textrm{eff}} = \langle \textbf{f}^{(0)}\rangle _m+ \langle \textbf{f}^{(0)}\rangle _s, \end{aligned}$$

69b $$\begin{aligned}&\mathsf T_{\textrm{eff}} =\left( \langle \mathbb {C}_m\mathbb {L}_m+\mathbb {C}_m\rangle _m +\langle \mathbb {C}_s\mathbb {L}_s+\mathbb {C}_s\rangle _s\right) \zeta _x(\textbf{u}^{(0)}) +\left\langle \mathbb {C}_m\boldsymbol{\tau }_m-\boldsymbol{\alpha }_m\right\rangle _m p_m^{(0)}\nonumber \\&~~~~~\quad +\left\langle \mathbb {C}_s\boldsymbol{\tau }_s-\boldsymbol{\alpha }_s\right\rangle _s p_s^{(0)} +\left\langle \mathbb {C}_m\tilde{\boldsymbol{\tau }}_m\right\rangle _m p_s^{(0)} +\left\langle \mathbb {C}_s\tilde{\boldsymbol{\tau }}_s\right\rangle _s p_m^{(0)} +\left\langle \mathbb {C}_m\boldsymbol{\theta }_m\right\rangle _m\nonumber \\&~~~~~\quad +\left\langle \mathbb {C}_s\boldsymbol{\theta }_s\right\rangle _s , \end{aligned}$$

69c $$\begin{aligned}&\mathcal {A}_m\dot{p}_m^{(0)}+ \mathcal {A}_s\dot{p}_s^{(0)} = -\nabla _x\cdot \textbf{w}_{\textrm{eff}} -\bar{\boldsymbol{\alpha }}:\zeta _x(\dot{\textbf{u}}^{(0)}), \end{aligned}$$

69d $$\begin{aligned}&\textbf{w}_{\textrm{eff}} = -{\mathsf W}\nabla _x\bar{p} -\langle \mathsf K_m\nabla _x\hat{p}_m\rangle _m -\langle \mathsf K_s\nabla _x\hat{p}_s\rangle _s -\langle \mathsf K_m\textbf{t}_m\rangle _m -\langle \mathsf K_s\textbf{t}_s\rangle _s +\textbf{F}_{L}. \end{aligned}$$

Here W is defined in (53), $\textbf{F}_L$ in (54), and $\textbf{f}^{(0)}$ in (62).

This specialisation highlights a key distinction between generic body forces and forces possessing gradient or curl structure. In the Lorentz case, the forcing does not simply enter the homogenised equations as an averaged source term. It also modifies the effective pressure and flow relations through additional corrector contributions, thereby altering the effective constitutive behaviour of the double-poroelastic medium.

## Comparison Between the General Force Model and the Magnetic Lorentz Force Model

We now want to compare the expressions obtained in the general macroscale model derived in Sect. 3.6 with the Lorentz force model derived in Sect. 4.4.

We begin by considering Darcy’s law, and compare (69d) with the general expression (40d). For convenience we restate (40d) as

70 $$\begin{aligned} \textbf{w}_{\textrm{eff}}=-{\textsf{W}}\nabla _x {p}^{(0)}-\langle {\textsf{K}}_{m}  \textbf{s}_{m}\rangle _m+\langle \textsf{K}_{m}\textbf{f}_{mf}^{(0)}\rangle _m\ -\langle {\textsf{K}}_{s}  \textbf{s}_{s}\rangle _s+\langle \textsf{K}_{s}\textbf{f}_{sf}^{(0)}\rangle _s \end{aligned}$$

where

71 $$\begin{aligned} {{\textbf {f}}}_{mf} = -\textsf{G}_m \nabla \phi _m \times {\textbf {B}} \quad \text{ and } \quad {{\textbf {f}}}_{sf} = -\textsf{G}_s \nabla \phi _s \times {\textbf {B}} \end{aligned}$$

Applying the multiple scales expansion we obtain

72 $$\begin{aligned} \epsilon {{\textbf {f}}}_{mf}^{\epsilon } = -\textsf{G}_m \nabla _y \phi _m^{\epsilon } \times {\textbf {B}}^{\epsilon }-\epsilon \textsf{G}_m \nabla _x \phi _m^{\epsilon } \times {\textbf {B}}^{\epsilon }\end{aligned}$$

73 $$\begin{aligned} \epsilon {{\textbf {f}}}_{sf}^{\epsilon } = -\textsf{G}_s\nabla _y \phi _s^{\epsilon } \times {\textbf {B}}^{\epsilon }-\epsilon \textsf{G}_s \nabla _x \phi _s^{\epsilon } \times {\textbf {B}}^{\epsilon } \end{aligned}$$

We can equate the powers of ϵ in these two forces. For $\epsilon ^0$ we obtain

74 $$\begin{aligned} -\textsf{G}_m \nabla _y \phi _m^{(0)} \times {\textbf {B}}^{(0)}=0 \quad \text{ and }\quad -\textsf{G}_s\nabla _y \phi _s^{(0)} \times {\textbf {B}}^{(0)}=0 \end{aligned}$$

and the coefficients of $\epsilon ^1$ give

75 $$\begin{aligned} {{\textbf {f}}}_{mf}^{(0)} = -\textsf{G}_m \nabla _y \phi _m^{(1)} \times {\textbf {B}}^{(0)}-\textsf{G}_m \nabla _y \phi _m^{(0)} \times {\textbf {B}}^{(1)}-\textsf{G}_m \nabla _x \phi _m^{(0)} \times {\textbf {B}}^{(0)} \end{aligned}$$

76 $$\begin{aligned} {{\textbf {f}}}_{sf}^{(0)} = -\textsf{G}_s \nabla _y \phi _s^{(1)} \times {\textbf {B}}^{(0)}-\textsf{G}_s \nabla _y \phi _s^{(0)} \times {\textbf {B}}^{(1)}-\textsf{G}_s \nabla _x \phi _s^{(0)} \times {\textbf {B}}^{(0)} \end{aligned}$$

We can substitute these expressions into (70) to obtain

77 $$\begin{aligned} \textbf{w}_{\textrm{eff}}&=-{\textsf{W}}\nabla _x {p}^{(0)}-\langle \textsf{K}_m \textbf{s}_m\rangle _m - \langle \textsf{K}_s \textbf{s}_s \rangle _s +\langle \textsf{K}_m(\textsf{G}_m\nabla _x \phi _m^{(0)} \times {\textbf {B}}^{(0)}) \rangle _m \nonumber \\&\qquad +\langle \textsf{K}_s(\textsf{G}_s \nabla _x \phi _s^{(0)} \times {\textbf {B}}^{(0)} )\rangle _s+\langle \textsf{K}_m(\textsf{G}_m\nabla _y\phi _m^{(0)} \times {\textbf {B}}^{(1)})\rangle _m \nonumber \\&\qquad +\langle \textsf{K}_s(\textsf{G}_s \nabla _y\phi _s^{(0)} \times {\textbf {B}}^{(1)})\rangle _s + \langle \textsf{K}_m(\textsf{G}_m \nabla _y\phi _m^{(1)} \times {\textbf {B}}^{(0)})\rangle _m\nonumber \\&\qquad + \langle \textsf{K}_s(\textsf{G}_s \nabla _y\phi _s^{(1)} \times {\textbf {B}}^{(0)})\rangle _s \end{aligned}$$

Comparing (77) and (69d), we see that (69d) differs from the generic Darcy law (77) because, in the Lorentz case, the body force is generated by electrical currents of the form $\textbf{j}_\alpha = -\mathbb {G}_\alpha \nabla \phi _\alpha$, α=m,s and therefore depends explicitly on the gradient of an electromagnetic potential. When this structure is carried through the homogenisation procedure, the curl-free component of the Lorentz force can be rewritten as the gradient of an additional *magnetic pressure*. This produces the extra pressure-type contribution that appears in (69d), while the remaining non–curl-free component enters as a standard forcing term, in direct analogy with the generic body-force contributions already present in (77).

By contrast, if the chosen body force does not contain a gradient, divergence, or any structure that can be recast as a potential, then no such decomposition occurs. Most classical body forces in poroelasticity such as gravity, buoyancy, or drag enter the homogenised Darcy law purely as source terms and do not modify the pressure-gradient structure. The magnetic Lorentz force is exceptional because its dependence on $\nabla \phi _\alpha$, α=m,s allows part of its homogenised contribution to behave exactly like a pressure gradient at the macroscale.

The stress balance equations (40a) and (69a) will coincide when the leading order magnetic Lorentz force is used in (40a). However, the influence of magnetic forcing appears in the effective stress tensor (69b) where the leading order pressure is no longer unified.

This lack of unified leading order pressure also influences the conservation of mass equation (69c). Comparing with (40c) we can see that Lorentz effects enter via the effective flux and via the Biot’s moduli.

## Conclusion

In this work, we have developed a rigorous multiscale framework for determining the effective behaviour of materials composed of a poroelastic matrix containing embedded poroelastic subphases, each governed by the fully anisotropic and heterogeneous Biot equations and each subject to body forces. By applying the asymptotic homogenisation technique [29, 30, 44], under assumptions of scale separation, periodic microstructure, and macroscopic uniformity, we derived a closed system of Biot-type equations that captures the coupled mechanical and fluid-transport response of the composite at the macroscale.

Unlike existing double-poroelastic and poroelastic composite models, the present framework explicitly incorporates body forces acting at the mesoscale and tracks their influence through to the effective macroscale equations. The resulting effective coefficients capture not only elastic and hydraulic heterogeneity, but also force-induced couplings that are absent from the classical Biot theory.

A central outcome of the analysis is the explicit form of the effective stress tensor, hydraulic conductivity tensor, and Biot-type coupling coefficients, each obtained from well-posed cell problems on the representative microstructural domain. These quantities reveal how mesoscale interactions between matrix and subphases shape the macroscale poromechanical response, and they generalise existing models for single-phase, composite, and double-poroelastic materials [34, 35, 38].

By specialising the framework to the case of magnetic Lorentz forcing, we demonstrated that body forces possessing a gradient structure lead to qualitatively different effective behaviour. In particular, such forces generate additional pressure-like contributions in the homogenised Darcy law, rather than appearing solely as averaged source terms. This highlights the importance of correctly accounting for the microscale structure of applied forces when modelling coupled poroelastic systems.

The framework presented here provides a foundation for analysing a broad class of multiphase poroelastic materials, including biological tissues, engineered composites, and geomechanical systems with embedded inclusions or fibres. The present analysis relies on several simplifying assumptions, including mesoscale periodicity, macroscopic uniformity of the microstructure, and quasi-static, linear poroelastic behaviour. While these assumptions enable a tractable and rigorous derivation, relaxing them would allow the framework to capture a wider range of physically relevant phenomena.

A natural extension of the present work is the relaxation of the macroscopic uniformity assumption [23], allowing the mesoscale geometry and material coefficients to vary slowly in space. In such locally periodic settings, additional transport and drift terms arise in the homogenised equations, reflecting the evolution of the underlying microstructure.

Extending the framework to nonlinear poroelastic constitutive laws or evolving microstructures [36, 40] and modelling microstructural alterations such as those induced by disease or remodelling [39] would require the solution of parameter-dependent cell problems and the development of consistent update strategies for the effective coefficients, posing both analytical and computational challenges. These challenges make the resulting cell problems computationally expensive to solve. Emerging techniques in the literature aim to overcome this issue, for example those in [49, 50], making extensions of this kind more computationally feasible.

This work could be extended to account for discontinuous volume loads governing the balance of linear momentum of the poroelastic phases by adapting the formulation for electrostrictive composites [37], in which forces can be written as the divergence of suitable inelastic stress components, such as the Maxwell stress tensor.

From a computational perspective, the explicit characterisation of the effective coefficients through cell problems facilitates numerical implementation using finite element or reduced-order methods. Developing efficient solution strategies, particularly in three dimensions and for highly anisotropic materials, remains an important direction for future research. Extending these computational approaches to realistic geometries, including biological imaging, would further enable quantitative predictions for application-specific scenarios.

From a biological perspective, Lorentz forces generated during MRI arise from the interaction between induced ionic currents and the applied magnetic field and are known to produce measurable tissue displacements (e.g., [51, 52]). In a double-poroelastic tissue, where intracellular (subphase) and extracellular (matrix) compartments may differ in electrical conductivity and hydraulic properties, such forces would not be distributed uniformly at the mesoscale. This suggests that MRI-induced electromagnetic forcing could drive compartment-specific pressure variations and relative fluid motion, potentially modulating perfusion, interstitial transport, or mechanotransductive signalling. Consequently, the multiscale coupling identified in our analysis may provide a mechanistic link between electromagnetic contrasts and measurable poromechanical responses in heterogeneous biological tissues.

## Data Availability

No datasets were generated or analysed during the current study.

## Appendix A Technical Details of the Homogenisation Derivation

This appendix contains the technical details supporting the homogenisation procedure presented in Sect. 3. The main text focuses on the key cell problems, effective coefficients, and resulting macroscale equations, while the order-by-order expansions and intermediate averaging steps are collected here for completeness. This organisation preserves the reproducibility of the derivation while keeping the main presentation focused on the structure and interpretation of the effective model.

### A.1 Order-by-Order Expansion of the Two-Scale Problem

In this appendix we provide the order-by-order details of the two-scale expansion used in Sect. 3. Applying the transformations (11) and (12) to the dimensionless system (8a)–(8l), and using the periodicity of the representative cell, gives

A1a $$\begin{aligned} \epsilon \nabla _\textbf{x}\cdot \textsf{T}_m^\epsilon +\nabla _\textbf{y}\cdot \textsf{T}_m^\epsilon =\epsilon \textbf{f}_{m}^\epsilon&\qquad \text{ in } \quad \Omega _{m},\end{aligned}$$

A1b $$\begin{aligned} \epsilon \nabla _\textbf{x}\cdot \textsf{T}_s^\epsilon +\nabla _\textbf{y}\cdot \textsf{T}_s^\epsilon =\epsilon \textbf{f}_{s}^\epsilon&\qquad \text{ in } \quad \Omega _{s},\end{aligned}$$

A1c $$\begin{aligned} \epsilon {\textsf{T}}_{m}^\epsilon =\mathbb {C}_{m}:\zeta _y (\textbf{u}_{m}^\epsilon )+\epsilon \mathbb {C}_{m}:\zeta _x (\textbf{u}_{m}^\epsilon )-\epsilon {\boldsymbol{\alpha }}_{m}p_{m}^\epsilon&\qquad \text{ in } \quad \Omega _{m},\end{aligned}$$

A1d $$\begin{aligned} \epsilon {\textsf{T}}_{s}^\epsilon =\mathbb {C}_{s}:\zeta _y (\textbf{u}_{s}^\epsilon )+\epsilon \mathbb {C}_{s}:\zeta _x( \textbf{u}_{s}^\epsilon )-\epsilon {\boldsymbol{\alpha }}_{s}p_{s}^\epsilon&\qquad \text{ in } \quad \Omega _{s},\end{aligned}$$

A1e $$\begin{aligned} \epsilon \textbf{w}_{m}^\epsilon =-{\textsf{K}}_{m}\nabla _y p_{m}^\epsilon -\epsilon {\textsf{K}}_{m}\nabla _x p_{m}^\epsilon +\epsilon \textsf{K}_m\textbf{f}_{mf}&\qquad \text{ in } \quad \Omega _{m},\end{aligned}$$

A1f $$\begin{aligned} \epsilon \textbf{w}_{s}^\epsilon =-{\textsf{K}}_{s}\nabla _y p_{s}^\epsilon -\epsilon {\textsf{K}}_{s}\nabla _x p_{s}^\epsilon +\epsilon \textsf{K}_s\textbf{f}_{sf}&\qquad \text{ in } \quad \Omega _{s},\end{aligned}$$

A1g $$\begin{aligned} \epsilon \frac{\dot{p}_{m}^\epsilon }{{M}_{m}}=-{\boldsymbol{\alpha }}_{m}:\zeta _y ( \dot{\textbf{u}}_{m}^\epsilon )-\epsilon {\boldsymbol{\alpha }}_{m}:\zeta _x (\dot{\textbf{u}}_{m}^\epsilon )-\nabla _y\cdot \textbf{w}_{m}^\epsilon -\epsilon \nabla _x\cdot \textbf{w}_{m}^\epsilon&\qquad \text{ in } \quad \Omega _{m},\end{aligned}$$

A1h $$\begin{aligned} \epsilon \frac{\dot{p}_{s}^\epsilon }{{M}_{s}}=-{\boldsymbol{\alpha }}_{s}:\zeta _y (\dot{\textbf{u}}_{s}^\epsilon )-\epsilon {\boldsymbol{\alpha }}_{s}:\zeta _x (\dot{\textbf{u}}_{s}^\epsilon )-\nabla _y\cdot \textbf{w}_{s}^\epsilon -\epsilon \nabla _x\cdot \textbf{w}_{s}^\epsilon&\qquad \text{ in } \quad \Omega _{\textrm{s}},\end{aligned}$$

A1i $$\begin{aligned} {\textsf{T}}_{m}^\epsilon \textbf{n}={\textsf{T}}_{s}^\epsilon \textbf{n}&\qquad \text{ on } \quad \Gamma ,\end{aligned}$$

A1j $$\begin{aligned} \textbf{u}_{m}^\epsilon =\textbf{u}_{s}^\epsilon&\qquad \text{ on } \quad \Gamma ,\end{aligned}$$

A1k $$\begin{aligned} \textbf{w}_{m}^\epsilon \cdot \textbf{n}=\textbf{w}_{s}^\epsilon \cdot \textbf{n}&\qquad \text{ on } \quad \Gamma ,\end{aligned}$$

A1l $$\begin{aligned} p_{m}^\epsilon =p_{s}^\epsilon&\qquad \text{ on } \quad \Gamma . \end{aligned}$$

Substituting expansions of the form (12) into (A1a)–(A1l) and equating terms with equal powers of ϵ gives the hierarchy of problems used in Sect. 3 to derive the effective model.

For fields that retain a dependence on the mesoscale variable, we use the phase average

A2 $$\begin{aligned} \langle \varphi \rangle _\alpha = \frac{1}{|\Omega |} \int _{\Omega _\alpha }\varphi (\textbf{x},\textbf{y},t)\,d\textbf{y}, \qquad \alpha =m,s, \end{aligned}$$

where the integral is taken over one representative periodic cell and $|\Omega |=|\Omega _m|+|\Omega _s|$. We also define the average over the full cell by

A3 $$\begin{aligned} \langle \varphi _{m}+\varphi _{s} \rangle _{\scriptscriptstyle \Omega } = \frac{1}{|\Omega |} \left( \int _{\Omega _{m}} \varphi _{m}(\textbf{x}, \textbf{y}, t)\,d\textbf{y} + \int _{\Omega _{s}} \varphi _{s}(\textbf{x}, \textbf{y}, t)\,d\textbf{y} \right) . \end{aligned}$$

Equating terms of order $\epsilon ^0$ gives

A4a $$\begin{aligned} \nabla _\textbf{y}\cdot \textsf{T}_m^{(0)}=0&\qquad \text{ in }\quad \Omega _m,\end{aligned}$$

A4b $$\begin{aligned} \nabla _\textbf{y}\cdot \textsf{T}_s^{(0)}=0&\qquad \text{ in }\quad \Omega _s,\end{aligned}$$

A4c $$\begin{aligned} \mathbb {C}_{m}:\zeta _y (\textbf{u}_{m}^{(0)})=0&\qquad \text{ in }\quad \Omega _m,\end{aligned}$$

A4d $$\begin{aligned} \mathbb {C}_{s}:\zeta _y (\textbf{u}_{s}^{(0)})=0&\qquad \text{ in }\quad \Omega _s,\end{aligned}$$

A4e $$\begin{aligned} {\textsf{K}}_{m}\nabla _y p_{m}^{(0)}=0&\qquad \text{ in }\quad \Omega _m,\end{aligned}$$

A4f $$\begin{aligned} {\textsf{K}}_{s}\nabla _y p_{s}^{(0)}=0&\qquad \text{ in }\quad \Omega _s,\end{aligned}$$

A4g $$\begin{aligned} {\boldsymbol{\alpha }}_{m}:\zeta _y (\dot{\textbf{u}}_{m}^{(0)})+\nabla _y\cdot \textbf{w}_{m}^{(0)}=0&\qquad \text{ in }\quad \Omega _m,\end{aligned}$$

A4h $$\begin{aligned} {\boldsymbol{\alpha }}_{s}:\zeta _y (\dot{\textbf{u}}_{s}^{(0)})+\nabla _y\cdot \textbf{w}_{s}^{(0)}=0&\qquad \text{ in }\quad \Omega _s, \end{aligned}$$

A4i $$\begin{aligned} {\textsf{T}}_{m}^{(0)}  \textbf{n}={\textsf{T}}_{s}^{(0)}  \textbf{n}&\qquad \text{ on }\quad \Gamma , \end{aligned}$$

A4j $$\begin{aligned} \textbf{u}_{m}^{(0)}=\textbf{u}_{s}^{(0)}&\qquad \text{ on }\quad \Gamma ,\end{aligned}$$

A4k $$\begin{aligned} \textbf{w}_{m}^{(0)}\cdot \textbf{n}=\textbf{w}_{s}^{(0)}\cdot \textbf{n}&\qquad \text{ on }\quad \Gamma ,\end{aligned}$$

A4l $$\begin{aligned} p_{m}^{(0)}=p_{s}^{(0)}&\qquad \text{ on }\quad \Gamma . \end{aligned}$$

Equations (A4c) and (A4d), together with periodicity, imply that the leading-order displacements are independent of the mesoscale variable. Hence

A5a $$\begin{aligned} \textbf{u}_m^{(0)}&=\textbf{u}_m^{(0)}(\textbf{x},t),\end{aligned}$$

A5b $$\begin{aligned} \textbf{u}_s^{(0)}&=\textbf{u}_s^{(0)}(\textbf{x},t). \end{aligned}$$

The interface condition (A4j) then gives

A6 $$\begin{aligned} \textbf{u}^{(0)}=\textbf{u}_m^{(0)}=\textbf{u}_s^{(0)}. \end{aligned}$$

Similarly, (A4e) and (A4f), together with (A4l), imply that the leading-order pressure is independent of $\textbf{y}$ and common to both phases:

A7 $$\begin{aligned} p^{(0)}=p_m^{(0)}(\textbf{x}, t)=p_s^{(0)}(\textbf{x}, t). \end{aligned}$$

Equating terms of order $\epsilon ^1$ gives

A8a $$\begin{aligned} \nabla _\textbf{x}\cdot \textsf{T}_m^{(0)}+\nabla _\textbf{y}\cdot \textsf{T}_m^{(1)}= \textsf{f}_{m}^{(0)}&\qquad \text{ in }\quad \Omega _m,\end{aligned}$$

A8b $$\begin{aligned} \nabla _\textbf{x}\cdot \textsf{T}_s^{(0)}+\nabla _\textbf{y}\cdot \textsf{T}_s^{(1)}=\textsf{f}_{s}^{(0)}&\qquad \text{ in }\quad \Omega _s,\end{aligned}$$

A8c $$\begin{aligned} {\textsf{T}}_{m}^{(0)}=\mathbb {C}_{m}:\zeta _y (\textbf{u}_{m}^{(1)})+\mathbb {C}_{m}:\zeta _x (\textbf{u}_{m}^{(0)})-{\boldsymbol{\alpha }}_{m}p_{m}^{(0)}&\qquad \text{ in }\quad \Omega _m,\end{aligned}$$

A8d $$\begin{aligned} {\textsf{T}}_{s}^{(0)}=\mathbb {C}_{s}:\zeta _y (\textbf{u}_{s}^{(1)})+\mathbb {C}_{s}:\zeta _x( \textbf{u}_{s}^{(0)})-{\boldsymbol{\alpha }}_{s}p_{s}^{(0)}&\qquad \text{ in }\quad \Omega _s, \end{aligned}$$

A8e $$\begin{aligned} \textbf{w}_{m}^{(0)}=-{\textsf{K}}_{m}\nabla _y p_{m}^{(1)}-{\textsf{K}}_{m}\nabla _x p_{m}^{(0)}+\textsf{K}_m\textbf{f}_{mf}^{(0)}&\qquad \text{ in }\quad \Omega _m,\end{aligned}$$

A8f $$\begin{aligned} \textbf{w}_{s}^{(0)}=-{\textsf{K}}_{s}\nabla _y p_{s}^{(1)}-{\textsf{K}}_{s}\nabla _x p_{s}^{(0)}+\textsf{K}_s\textbf{f}_{sf}^{(0)}&\qquad \text{ in }\quad \Omega _s,\end{aligned}$$

A8g $$\begin{aligned} \frac{\dot{p}_{m}^{(0)}}{{M}_{m}}=-{\boldsymbol{\alpha }}_{m}:\zeta _y (\dot{\textbf{u}}_{m}^{(1)})-{\boldsymbol{\alpha }}_{m}:\zeta _x ( \dot{\textbf{u}}_{m}^{(0)})-\nabla _y\cdot \textbf{w}_{m}^{(1)}-\nabla _x\cdot \textbf{w}_{m}^{(0)}&\qquad \text{ in }\quad \Omega _m,\end{aligned}$$

A8h $$\begin{aligned} \frac{\dot{p}_{s}^{(0)}}{{M}_{s}}=-{\boldsymbol{\alpha }}_{s}:\zeta _y (\dot{\textbf{u}}_{s}^{(1)})-{\boldsymbol{\alpha }}_{s}:\zeta _x ( \dot{\textbf{u}}_{s}^{(0)})-\nabla _y\cdot \textbf{w}_{s}^{(1)}-\nabla _x\cdot \textbf{w}_{s}^{(0)}&\qquad \text{ in }\quad \Omega _s,\end{aligned}$$

A8i $$\begin{aligned} {\textsf{T}}_{m}^{(1)}  \textbf{n}={\textsf{T}}_{s}^{(1)}  \textbf{n}&\qquad \text{ on }\quad \Gamma ,\end{aligned}$$

A8j $$\begin{aligned} \textbf{u}_{m}^{(1)}=\textbf{u}_{s}^{(1)}&\qquad \text{ on }\quad \Gamma ,\end{aligned}$$

A8k $$\begin{aligned} \textbf{w}_{m}^{(1)}\cdot \textbf{n}=\textbf{w}_{s}^{(1)}\cdot \textbf{n}&\qquad \text{ on }\quad \Gamma ,\end{aligned}$$

A8l $$\begin{aligned} p_{m}^{(1)}=p_{s}^{(1)}&\qquad \text{ on }\quad \Gamma . \end{aligned}$$

These $\epsilon ^0$ and $\epsilon ^1$ problems are the starting point for the cell problems for the displacement correctors, pressure correctors, and effective fluxes derived in Sect. 3.

### Averaging of the Momentum Balance

For completeness, we provide the averaging step used to obtain the effective momentum balance in Sect. 3.3. Integrating the $\epsilon ^1$ momentum Eqs.  (A8a) and (A8b) over the matrix and subphase domains gives

A9 $$\begin{aligned}&\int _{\Omega _m}\nabla _\textbf{x}\cdot \textsf{T}_m^{(0)}\,\textrm{d}  \textbf{y} +\int _{\Omega _m}\nabla _\textbf{y}\cdot \textsf{T}_m^{(1)}\,\textrm{d}  \textbf{y} +\int _{\Omega _s}\nabla _\textbf{x}\cdot \textsf{T}_s^{(0)}\,\textrm{d}  \textbf{y} +\int _{\Omega _s}\nabla _\textbf{y}\cdot \textsf{T}_s^{(1)}\,\textrm{d}  \textbf{y} \nonumber \\&\qquad -\int _{\Omega _m} \textbf{f}_m^{(0)}\,\textrm{d}  \textbf{y} -\int _{\Omega _s} \textbf{f}_s^{(0)}\,\textrm{d}  \textbf{y} =0 . \end{aligned}$$

Using macroscopic uniformity, the $\nabla _\textbf{x}$ derivatives may be taken outside the cell integrals. Applying the divergence theorem to the $\nabla _\textbf{y}$ terms yields

A10 $$\begin{aligned}&\nabla _\textbf{x}\cdot \langle \textsf{T}_m^{(0)}\rangle _m +\nabla _\textbf{x}\cdot \langle \textsf{T}_s^{(0)}\rangle _s +\int _{\partial \Omega _m\setminus \Gamma } \textsf{T}_m^{(1)}  \textbf{n}_{\partial \Omega _m}\,\textrm{d}S -\int _{\Gamma } \textsf{T}_m^{(1)}  \textbf{n}\,\textrm{d}S \nonumber \\&\qquad +\int _{\partial \Omega _s\setminus \Gamma } \textsf{T}_s^{(1)}  \textbf{n}_{\partial \Omega _s}\,\textrm{d}S +\int _{\Gamma } \textsf{T}_s^{(1)}  \textbf{n}\,\textrm{d}S -\langle \textbf{f}_m^{(0)}\rangle _m -\langle \textbf{f}_s^{(0)}\rangle _s =0 . \end{aligned}$$

The boundary contributions on $\partial \Omega _m\setminus \Gamma$ and $\partial \Omega _s\setminus \Gamma$ cancel by periodicity. The interface terms on Γ cancel by the traction-continuity condition (A8i). Hence,

A11 $$\begin{aligned} \nabla _\textbf{x}\cdot \langle \textsf{T}_m^{(0)}\rangle _m + \nabla _\textbf{x}\cdot \langle \textsf{T}_s^{(0)}\rangle _s = \langle \textbf{f}_m^{(0)}\rangle _m + \langle \textbf{f}_s^{(0)}\rangle _s . \end{aligned}$$

Defining

A12 $$\begin{aligned} \textsf{T}_{\textrm{eff}} = \langle \textsf{T}_m^{(0)}\rangle _m + \langle \textsf{T}_s^{(0)}\rangle _s , \end{aligned}$$

we obtain the effective momentum balance

A13 $$\begin{aligned} \nabla _\textbf{x}\cdot \textsf{T}_{\textrm{eff}} = \langle \textbf{f}_m^{(0)}\rangle _m + \langle \textbf{f}_s^{(0)}\rangle _s . \end{aligned}$$

### A.2 Averaging of the Conservation of Mass Equation

For completeness, we provide the averaging step used to obtain the effective conservation of mass equation in Sect. 3.5. Integrating the $\epsilon ^1$ mass balance equations (A8g) and (A8h) over $\Omega _m$ and $\Omega _s$ gives

A14 $$\begin{aligned}&\int _{\Omega _{m}}\frac{\dot{p}^{(0)}}{{M}_{m}}\,\textrm{d}\textbf{y} +\int _{\Omega _{s}}\frac{\dot{p}^{(0)}}{{M}_{s}}\,\textrm{d}\textbf{y} = -\int _{\Omega _{m}} \boldsymbol{\alpha }_{m}:\zeta _x(\dot{\textbf{u}}^{(0)})\,\textrm{d}\textbf{y} -\nabla _x \cdot \int _{\Omega _{m}}  \textbf{w}_{m}^{(0)}\,\textrm{d}\textbf{y} \nonumber \\&\quad -\int _{\Omega _{s}} \boldsymbol{\alpha }_{s}:\zeta _x(\dot{\textbf{u}}^{(0)})\,\textrm{d}\textbf{y} -\nabla _x \cdot \int _{\Omega _{s}}  \textbf{w}_{s}^{(0)}\,\textrm{d}\textbf{y} -\int _{\Omega _{m}} \boldsymbol{\alpha }_{m}:\zeta _y(\dot{\textbf{u}}_{m}^{(1)})\,\textrm{d}\textbf{y} \nonumber \\&\quad -\int _{\Omega _{s}} \boldsymbol{\alpha }_{s}:\zeta _y(\dot{\textbf{u}}_{s}^{(1)})\,\textrm{d}\textbf{y} -\int _{\Omega _{m}} \nabla _y\cdot \textbf{w}_{m}^{(1)}\,\textrm{d}\textbf{y} -\int _{\Omega _{s}} \nabla _y\cdot \textbf{w}_{s}^{(1)}\,\textrm{d}\textbf{y}. \end{aligned}$$

Applying the divergence theorem to the final two terms, using periodicity on the external cell boundary and the flux-continuity condition (A8k) on Γ, these boundary contributions cancel. Hence,

A15 $$\begin{aligned} \bigg ( \frac{\langle {M}_{m}+{M}_{s}\rangle _{\scriptscriptstyle \Omega }}{\langle {M}_{m}\rangle _{m}\langle {M}_{s}\rangle _{s}} \bigg )\dot{p}^{(0)}&= -\langle \boldsymbol{\alpha }_{m}+\boldsymbol{\alpha }_{s}\rangle _{\scriptscriptstyle \Omega } :\zeta _x(\dot{\textbf{u}}^{(0)}) -\nabla _x\cdot \langle \textbf{w}_{m}^{(0)}+\textbf{w}_{s}^{(0)}\rangle _{\scriptscriptstyle \Omega } \nonumber \\&\quad -\left\langle \boldsymbol{\alpha }_{m}:\zeta _y(\dot{\textbf{u}}_{m}^{(1)}) + \boldsymbol{\alpha }_{s}:\zeta _y(\dot{\textbf{u}}_{s}^{(1)}) \right\rangle _{\scriptscriptstyle \Omega }. \end{aligned}$$

Using the first-order displacement correctors (15a)–(15b), we obtain

A16 $$\begin{aligned}&\bigg ( \frac{\langle {M}_{m}+{M}_{s}\rangle _{\scriptscriptstyle \Omega }}{\langle {M}_{m}\rangle _{m}\langle {M}_{s}\rangle _{s}} \bigg )\dot{p}^{(0)} = -\bigg ( \langle \boldsymbol{\alpha }_{m}+\boldsymbol{\alpha }_{s}\rangle _{\scriptscriptstyle \Omega } :\zeta _x(\dot{\textbf{u}}^{(0)}) + \nabla _x\cdot \textbf{w}_{\textrm{eff}} \nonumber \\&\qquad + \left\langle \mathbb {L}_{m}^{\textrm{T}}:\boldsymbol{\alpha }_{m} + \mathbb {L}_{s}^{\textrm{T}}:\boldsymbol{\alpha }_{s} \right\rangle _{\scriptscriptstyle \Omega } :\zeta _x(\dot{\textbf{u}}^{(0)}) + \left\langle \boldsymbol{\alpha }_{m}:{\boldsymbol{\tau }}_{m} + \boldsymbol{\alpha }_{s}:{\boldsymbol{\tau }}_{s} \right\rangle _{\scriptscriptstyle \Omega } \dot{p}^{(0)} \bigg ). \end{aligned}$$

Rearranging (A16) gives the effective conservation of mass equation reported in Sect. 3.5.

## Appendix B Details of the Lorentz-Force Specialisation

This appendix contains the order-by-order calculations used to obtain the Lorentz-modified effective equations in Sect. 4. The main text retains the key Lorentz-induced cell problems and the final effective equations, while the repeated expansions, longer auxiliary cell problems, and intermediate averaging steps are collected here.

### B.1 Lorentz-Modified Darcy Flow

Starting from (43)–(44), the two-scale expansion gives

B1 $$\begin{aligned} \epsilon \textbf{w}_m^{\epsilon }&=-\textsf{K}_m\nabla _y p_m^{\epsilon } -\epsilon \textsf{K}_m\nabla _x p_m^{\epsilon } +\textsf{K}_m\textsf{G}_m\nabla _y\phi _m^{\epsilon }\times \textsf{B}^{\epsilon } +\epsilon \textsf{K}_m\textsf{G}_m\nabla _x\phi _m^{\epsilon }\times \textsf{B}^{\epsilon }, \end{aligned}$$

B2 $$\begin{aligned} \epsilon \textbf{w}_s^{\epsilon }&=-\textsf{K}_s\nabla _y p_s^{\epsilon } -\epsilon \textsf{K}_s\nabla _x p_s^{\epsilon } +\textsf{K}_s\textsf{G}_s\nabla _y\phi _s^{\epsilon }\times \textsf{B}^{\epsilon } +\epsilon \textsf{K}_s\textsf{G}_s\nabla _x\phi _s^{\epsilon }\times \textsf{B}^{\epsilon }. \end{aligned}$$

Using these expressions in the phase-wise conservation of mass equations and multiplying through by $\epsilon ^2$ gives

B3 $$\begin{aligned}&\epsilon ^2\frac{\dot{p}^{\epsilon }_m}{M_m} =-\epsilon \boldsymbol{\alpha }_m:\zeta _y(\dot{\textbf{u}}^{\epsilon }_m) -\epsilon ^2\boldsymbol{\alpha }_m:\zeta _x(\dot{\textbf{u}}^{\epsilon }_m) +\epsilon ^2 \nabla _x \cdot (\textsf{K}_m \nabla _x p_m ^\epsilon ) -\epsilon ^2\nabla _x \cdot (\textsf{K}_m(\textsf{G}_m\nabla _x \phi _m^\epsilon \times \textbf{B}^\epsilon )) \nonumber \\&\quad +\epsilon \nabla _y \cdot (\textsf{K}_m \nabla _x p_m ^\epsilon ) +\epsilon \nabla _x \cdot (\textsf{K}_m \nabla _y p_m ^\epsilon ) -\epsilon \nabla _x \cdot (\textsf{K}_m(\textsf{G}_m\nabla _y\phi _m^\epsilon \times \textbf{B}^\epsilon )) \nonumber \\&\quad +\nabla _y \cdot (\textsf{K}_m \nabla _y p_m ^\epsilon ) -\epsilon \nabla _y \cdot (\textsf{K}_m(\textsf{G}_m\nabla _x\phi _m^\epsilon \times \textbf{B}^\epsilon )) -\nabla _y \cdot (\textsf{K}_m(\textsf{G}_m\nabla _y\phi _m^\epsilon \times \textbf{B}^\epsilon )), \end{aligned}$$

B4 $$\begin{aligned}&\epsilon ^2\frac{\dot{p}^{\epsilon }_s}{M_s} =-\epsilon \boldsymbol{\alpha }_s:\zeta _y(\dot{\textbf{u}}^{\epsilon }_s) -\epsilon ^2\boldsymbol{\alpha }_s:\zeta _x(\dot{\textbf{u}}^{\epsilon }_s) +\epsilon ^2 \nabla _x \cdot (\textsf{K}_s \nabla _x p_s ^\epsilon ) -\epsilon ^2\nabla _x \cdot (\textsf{K}_s(\textsf{G}_s \nabla _x \phi _s^\epsilon \times \textbf{B}^\epsilon )) \nonumber \\&\quad +\epsilon \nabla _y \cdot (\textsf{K}_s \nabla _x p_s ^\epsilon ) +\epsilon \nabla _x \cdot (\textsf{K}_s \nabla _y p_s ^\epsilon ) -\epsilon \nabla _x \cdot (\textsf{K}_s(\textsf{G}_s \nabla _y\phi _s^\epsilon \times \textbf{B}^\epsilon )) \nonumber \\&\quad +\nabla _y \cdot (\textsf{K}_s \nabla _y p_s ^\epsilon ) -\epsilon \nabla _y \cdot (\textsf{K}_s(\textsf{G}_s \nabla _x\phi _s^\epsilon \times \textbf{B}^\epsilon )) -\nabla _y \cdot (\textsf{K}_s(\textsf{G}_s \nabla _y\phi _s^\epsilon \times \textbf{B}^\epsilon )). \end{aligned}$$

The corresponding flux and pressure interface conditions are

B5 $$\begin{aligned}&\left( \epsilon \textsf{K}_m\nabla _x p_m^\epsilon -\epsilon \mathsf K_m(\mathsf G_m\nabla _x\phi _m^\epsilon \times \textbf{B}^\epsilon ) +\mathsf K_m\nabla _y p_m^\epsilon -\mathsf K_m(\mathsf G_m\nabla _y\phi _m^\epsilon \times \textbf{B}^\epsilon )\right) \cdot \textbf{n} \nonumber \\&\qquad = \left( \epsilon \textsf{K}_s\nabla _x p_s^\epsilon -\epsilon \mathsf K_s(\mathsf G_s\nabla _x\phi _s^\epsilon \times \textbf{B}^\epsilon ) +\mathsf K_s\nabla _y p_s^\epsilon -\mathsf K_s(\mathsf G_s\nabla _y\phi _s^\epsilon \times \textbf{B}^\epsilon )\right) \cdot \textbf{n}, \end{aligned}$$

B6 $$\begin{aligned}&p_m^\epsilon =p_s^\epsilon \end{aligned}$$

Equating terms of order $\epsilon ^0$ gives the pressure problem (45a)–(45d). The decomposition (46)–(47) gives the pressure-corrector problem (48a)–(48d).

Equating terms of order $\epsilon ^1$ in (B3)–(B6) gives

B7a $$\begin{aligned}&\nabla _y\cdot (\mathsf K_m\nabla _x p_m^{(0)}) +\nabla _x\cdot (\mathsf K_m\nabla _y p_m^{(0)}) -\nabla _x\cdot (\mathsf K_m(\mathsf G_m\nabla _y\phi _m^{(0)}\times \textbf{B}^{(0)})) \nonumber \\&-\nabla _y\cdot (\mathsf K_m(\mathsf G_m\nabla _x\phi _m^{(0)}\times \textbf{B}^{(0)})) +\nabla _y\cdot (\mathsf K_m\nabla _y p_m^{(1)}) \nonumber \\&-\nabla _y\cdot (\mathsf K_m(\mathsf G_m\nabla _y\phi _m^{(0)}\times \textbf{B}^{(1)})) -\nabla _y\cdot (\mathsf K_m(\mathsf G_m\nabla _y\phi _m^{(1)}\times \textbf{B}^{(0)}))=0\nonumber \\&\quad \text{ in } \Omega _m, \end{aligned}$$

B7b $$\begin{aligned} \quad \nonumber \\&\nabla _y\cdot (\mathsf K_s\nabla _x p_s^{(0)}) +\nabla _x\cdot (\mathsf K_s\nabla _y p_s^{(0)}) -\nabla _x\cdot (\mathsf K_s(\mathsf G_s\nabla _y\phi _s^{(0)}\times \textbf{B}^{(0)})) \nonumber \\&-\nabla _y\cdot (\mathsf K_s(\mathsf G_s\nabla _x\phi _s^{(0)}\times \textbf{B}^{(0)})) +\nabla _y\cdot (\mathsf K_s\nabla _y p_s^{(1)}) \nonumber \\&-\nabla _y\cdot (\mathsf K_s(\mathsf G_s\nabla _y\phi _s^{(0)}\times \textbf{B}^{(1)})) -\nabla _y\cdot (\mathsf K_s(\mathsf G_s\nabla _y\phi _s^{(1)}\times \textbf{B}^{(0)}))=0\nonumber \\&\quad \text{ in } \Omega _s, \end{aligned}$$

B7c $$\begin{aligned} \quad \nonumber \\&\bigg ((\mathsf K_m\nabla _x p_m^{(0)} -\mathsf K_m(\mathsf G_m\nabla _x\phi _m^{(0)}\times \textbf{B}^{(0)}) +\mathsf K_m\nabla _y p_m^{(1)} -\mathsf K_m(\mathsf G_m\nabla _y\phi _m^{(0)}\times \textbf{B}^{(1)}) \nonumber \\&-\mathsf K_m(\mathsf G_m\nabla _y\phi _m^{(1)}\times \textbf{B}^{(0)})\bigg )\cdot \textbf{n} = \bigg (\mathsf K_s\nabla _x p_s^{(0)} -\mathsf K_s(\mathsf G_s\nabla _x\phi _s^{(0)}\times \textbf{B}^{(0)})+\mathsf K_s\nabla _y p_s^{(1)} \nonumber \\&-\mathsf K_s(\mathsf G_s\nabla _y\phi _s^{(0)}\times \textbf{B}^{(1)}) -\mathsf K_s(\mathsf G_s\nabla _y\phi _s^{(1)}\times \textbf{B}^{(0)})\bigg )\cdot \textbf{n} \quad \text{ on } \Gamma , \end{aligned}$$

B7d $$\begin{aligned}&p_m^{(1)}=p_s^{(1)}\quad \text{ on } \Gamma . \end{aligned}$$

The ansatz (49)–(50) leads to the cell problems for $\textbf{d}_m,\textbf{d}_s$:

B8a $$\begin{aligned}&\nabla _y\cdot (\nabla _y\textbf{d}_m\mathsf K_m^T)=-\nabla _y\cdot \mathsf K_m^T  &   \text{ in } \Omega _m,\end{aligned}$$

B8b $$\begin{aligned}&\nabla _y\cdot (\nabla _y\textbf{d}_s\mathsf K_s^T)=-\nabla _y\cdot \mathsf K_s^T  &   \text{ in } \Omega _s,\end{aligned}$$

B8c $$\begin{aligned}&\left( (\nabla _y\textbf{d}_m)\mathsf K_m^T-(\nabla _y\textbf{d}_s)\mathsf K_s^T\right) \textbf{n} =(\mathsf K_s-\mathsf K_m)^T\textbf{n}  &   \text{ on } \Gamma ,\end{aligned}$$

B8d $$\begin{aligned}&\textbf{d}_m=\textbf{d}_s  &   \text{ on } \Gamma . \end{aligned}$$

The scalar correctors $t_m,t_s$ solve

B9a $$\begin{aligned}&\nabla _y\cdot (\mathsf K_m\nabla _y t_m) +\nabla _y\cdot (\mathsf K_m\nabla _x\hat{p}_m) +\nabla _x\cdot (\mathsf K_m\nabla _y\hat{p}_m) -\nabla _x\cdot (\mathsf K_m(\mathsf G_m\nabla _y\phi _m^{(0)}\times \textbf{B}^{(0)})) \nonumber \\&-\nabla _y\cdot (\mathsf K_m(\mathsf G_m\nabla _x\phi _m^{(0)}\times \textbf{B}^{(0)})) -\nabla _y\cdot (\mathsf K_m(\mathsf G_m\nabla _y\phi _m^{(0)}\times \textbf{B}^{(1)}))\nonumber \\&-\nabla _y\cdot (\mathsf K_m(\mathsf G_m\nabla _y\phi _m^{(1)}\times \textbf{B}^{(0)}))=0 \quad \text{ in } \Omega _m,\end{aligned}$$

B9b $$\begin{aligned}&\nabla _y\cdot (\mathsf K_s\nabla _y t_s) +\nabla _y\cdot (\mathsf K_s\nabla _x\hat{p}_s) +\nabla _x\cdot (\mathsf K_s\nabla _y\hat{p}_s) -\nabla _x\cdot (\mathsf K_s(\mathsf G_s\nabla _y\phi _s^{(0)}\times \textbf{B}^{(0)})) \nonumber \\&-\nabla _y\cdot (\mathsf K_s(\mathsf G_s\nabla _x\phi _s^{(0)}\times \textbf{B}^{(0)})) -\nabla _y\cdot (\mathsf K_s(\mathsf G_s\nabla _y\phi _s^{(0)}\times \textbf{B}^{(1)}))\nonumber \\&-\nabla _y\cdot (\mathsf K_s(\mathsf G_s\nabla _y\phi _s^{(1)}\times \textbf{B}^{(0)}))=0\quad \text{ in } \Omega _s, \end{aligned}$$

B9c $$\begin{aligned}&\Big (\textsf{K}_m \nabla _y t_m+\textsf{K}_m\nabla _x \hat{p}_m - \textsf{K}_{m}(\textsf{G}_{m} \nabla _x\phi _{m}^{(0)} \times {\textbf {B}}^{(0)}) - \textsf{K}_{m}(\textsf{G}_{m} \nabla _y\phi _{m}^{(0)} \times {\textbf {B}}^{(1)}) \nonumber \\&-\textsf{K}_{m}(\textsf{G}_{m} \nabla _y\phi _{m}^{(1)} \times {\textbf {B}}^{(0)})\Big )\cdot {\textbf {n}} = \Big ( \textsf{K}_s \nabla _y t_s+\textsf{K}_s\nabla _x \hat{p}_s - \textsf{K}_s(\textsf{G}_s \nabla _x\phi _s^{(0)} \times {\textbf {B}}^{(0)}) \nonumber \\&- \textsf{K}_s(\textsf{G}_s \nabla _y\phi _s^{(0)} \times {\textbf {B}}^{(1)}) - \textsf{K}_s(\textsf{G}_s \nabla _y\phi _s^{(1)} \times {\textbf {B}}^{(0)})\Big )\cdot {\textbf {n}}\quad \text{ on } \Gamma ,\end{aligned}$$

B9d $$\begin{aligned}&t_m=t_s \quad \text{ on } \Gamma . \end{aligned}$$

These problems are supplemented with periodic boundary conditions and zero-average conditions.

The leading order relative fluid-solid velocities are

B10 $$\begin{aligned} \textbf{w}_m^{(0)}&=-\mathsf K_m\nabla _y p_m^{(1)} -\mathsf K_m\nabla _x p_m^{(0)} +\mathsf K_m\mathsf G_m\nabla _y\phi _m^{(1)}\times \mathsf B^{(0)} +\mathsf K_m\mathsf G_m\nabla _y\phi _m^{(0)}\times \mathsf B^{(1)} \nonumber \\&+\mathsf K_m\mathsf G_m\nabla _x\phi _m^{(0)}\times \mathsf B^{(0)}, \end{aligned}$$

B11 $$\begin{aligned} \textbf{w}_s^{(0)}&=-\mathsf K_s\nabla _y p_s^{(1)} -\mathsf K_s\nabla _x p_s^{(0)} +\mathsf K_s\mathsf G_s\nabla _y\phi _s^{(1)}\times \mathsf B^{(0)} +\mathsf K_s\mathsf G_s\nabla _y\phi _s^{(0)}\times \mathsf B^{(1)} \nonumber \\&+\mathsf K_s\mathsf G_s\nabla _x\phi _s^{(0)}\times \mathsf B^{(0)}. \end{aligned}$$

Substituting (46)–(50) and averaging gives

B12 $$\begin{aligned} \langle \textbf{w}_m^{(0)}\rangle _m&=-\langle \mathsf K_m\mathsf D_m+\mathsf K_m\rangle _m\nabla _x\bar{p} -\langle \mathsf K_m\textbf{t}_m\rangle _m -\langle \mathsf K_m\nabla _x\hat{p}_m\rangle _m \nonumber \\&\quad +\langle \mathsf K_m\mathsf G_m\nabla _y\phi _m^{(1)}\times \mathsf B^{(0)}\rangle _m +\langle \mathsf K_m\mathsf G_m\nabla _y\phi _m^{(0)}\times \mathsf B^{(1)}\rangle _m +\langle \mathsf K_m\mathsf G_m\nabla _x\phi _m^{(0)}\times \mathsf B^{(0)}\rangle _m, \end{aligned}$$

B13 $$\begin{aligned} \langle \textbf{w}_s^{(0)}\rangle _s&=-\langle \mathsf K_s\mathsf D_s+\mathsf K_s\rangle _s\nabla _x\bar{p} -\langle \mathsf K_s\textbf{t}_s\rangle _s -\langle \mathsf K_s\nabla _x\hat{p}_s\rangle _s \nonumber \\&\quad +\langle \mathsf K_s\mathsf G_s\nabla _y\phi _s^{(1)}\times \mathsf B^{(0)}\rangle _s +\langle \mathsf K_s\mathsf G_s\nabla _y\phi _s^{(0)}\times \mathsf B^{(1)}\rangle _s +\langle \mathsf K_s\mathsf G_s\nabla _x\phi _s^{(0)}\times \mathsf B^{(0)}\rangle _s. \end{aligned}$$

Adding (B12) and (B13) gives the effective Darcy law (52).

### B.2 Lorentz-Modified Poroelastic Cell Problems

The Lorentz-modified first-order poroelastic problem is

B14a $$\begin{aligned} \nabla _y \cdot (\mathbb {C}_m\zeta _y(\textbf{u}_m^{(1)}))&=-\nabla _y\cdot (\mathbb {C}_m\zeta _x(\textbf{u}^{(0)})) +\nabla _y\cdot (p_m^{(0)}\boldsymbol{\alpha }_m) -\mathsf G\nabla _y\phi ^{(0)}\times \mathsf B \quad \text{ in } \Omega _m,\end{aligned}$$

B14b $$\begin{aligned} \nabla _y \cdot (\mathbb {C}_s\zeta _y(\textbf{u}_s^{(1)}))&=-\nabla _y\cdot (\mathbb {C}_s\zeta _x(\textbf{u}^{(0)})) +\nabla _y\cdot (p_s^{(0)}\boldsymbol{\alpha }_s) -\mathsf G\nabla _y\phi ^{(0)}\times \mathsf B \quad \text{ in } \Omega _s,\end{aligned}$$

B14c $$\begin{aligned} (\mathbb {C}_m\zeta _y(\textbf{u}_m^{(1)})-\mathbb {C}_s\zeta _y(\textbf{u}_s^{(1)}))\textbf{n}&=((\mathbb {C}_s-\mathbb {C}_m)\zeta _x(\textbf{u}^{(0)})-\boldsymbol{\alpha }_s p_s^{(0)}+\boldsymbol{\alpha }_m p_m^{(0)})\textbf{n} \quad \text{ on } \Gamma ,\end{aligned}$$

B14d $$\begin{aligned} \textbf{u}_m^{(1)}&=\textbf{u}_s^{(1)} \quad \text{ on } \Gamma . \end{aligned}$$

The ansatz (56a)–(56b) leads to the following corrector problems. The elastic correctors solve

B15a $$\begin{aligned} \nabla _y \cdot (\mathbb {C}_m\zeta _y(\mathcal {B}_m))&=-\nabla _y\cdot \mathbb {C}_m  &   \text{ in } \Omega _m,\end{aligned}$$

B15b $$\begin{aligned} \nabla _y \cdot (\mathbb {C}_s\zeta _y(\mathcal {B}_s))&=-\nabla _y\cdot \mathbb {C}_s  &   \text{ in } \Omega _s,\end{aligned}$$

B15c $$\begin{aligned} (\mathbb {C}_m\zeta _y(\mathcal {B}_m)-\mathbb {C}_s\zeta _y(\mathcal {B}_s))\textbf{n}&=(\mathbb {C}_s-\mathbb {C}_m)\textbf{n}  &   \text{ on } \Gamma ,\end{aligned}$$

B15d $$\begin{aligned} \mathcal {B}_m&=\mathcal {B}_s  &   \text{ on } \Gamma . \end{aligned}$$

The pressure correctors associated with $p_m^{(0)}$ solve

B16a $$\begin{aligned} \nabla _y \cdot (\mathbb {C}_m\zeta _y(\textbf{b}_m))&=\nabla _y\cdot \boldsymbol{\alpha }_m  &   \text{ in } \Omega _m,\end{aligned}$$

B16b $$\begin{aligned} \nabla _y \cdot (\mathbb {C}_s\zeta _y(\tilde{\textbf{b}}_s))&=0  &   \text{ in } \Omega _s,\end{aligned}$$

B16c $$\begin{aligned} (\mathbb {C}_m\zeta _y(\textbf{b}_m)-\mathbb {C}_s\zeta _y(\tilde{\textbf{b}}_s))\textbf{n}&=\boldsymbol{\alpha }_m\textbf{n}  &   \text{ on } \Gamma ,\end{aligned}$$

B16d $$\begin{aligned} \textbf{b}_m&=\tilde{\textbf{b}}_s  &   \text{ on } \Gamma . \end{aligned}$$

The pressure correctors associated with $p_s^{(0)}$ solve

B17a $$\begin{aligned} \nabla _y \cdot (\mathbb {C}_m\zeta _y(\tilde{\textbf{b}}_m))&=0  &   \text{ in } \Omega _m,\end{aligned}$$

B17b $$\begin{aligned} \nabla _y \cdot (\mathbb {C}_s\zeta _y(\textbf{b}_s))&=\nabla _y\cdot \boldsymbol{\alpha }_s  &   \text{ in } \Omega _s,\end{aligned}$$

B17c $$\begin{aligned} (\mathbb {C}_m\zeta _y(\tilde{\textbf{b}}_m)-\mathbb {C}_s\zeta _y(\textbf{b}_s))\textbf{n}&=-\boldsymbol{\alpha }_s\textbf{n}  &   \text{ on } \Gamma ,\end{aligned}$$

B17d $$\begin{aligned} \tilde{\textbf{b}}_m&=\textbf{b}_s  &   \text{ on } \Gamma . \end{aligned}$$

The Lorentz-induced mechanical correctors are given in (57a)–(57d) in the main text. All these cell problems are supplemented with periodic boundary conditions on $\partial \Omega \setminus \Gamma$. The auxiliary fields are assigned zero average over their respective subdomains for uniqueness.

### B.3 Averaging of the Lorentz-Modified Conservation of Mass

Starting from the order $\epsilon ^1$ conservation of mass equations in $\Omega _m$ and $\Omega _s$, and retaining distinct leading-order pressures, we integrate over the two subdomains to obtain

B18 $$\begin{aligned}&\int _{\Omega _m}\frac{\dot{p}_m^{(0)}}{M_m}\,\mathrm d\textbf{y} +\int _{\Omega _s}\frac{\dot{p}_s^{(0)}}{M_s}\,\mathrm d\textbf{y} = -\int _{\Omega _m}\boldsymbol{\alpha }_m:\zeta _x(\dot{\textbf{u}}^{(0)})\,\mathrm d\textbf{y} -\nabla _x\cdot \int _{\Omega _m}  \textbf{w}_m^{(0)}\,\mathrm d\textbf{y} \nonumber \\&\quad -\int _{\Omega _s}\boldsymbol{\alpha }_s:\zeta _x(\dot{\textbf{u}}^{(0)})\,\mathrm d\textbf{y} -\nabla _x\cdot \int _{\Omega _s}  \textbf{w}_s^{(0)}\,\mathrm d\textbf{y} -\int _{\Omega _m}\boldsymbol{\alpha }_m:\zeta _y(\dot{\textbf{u}}_m^{(1)})\,\mathrm d\textbf{y} \nonumber \\&\quad -\int _{\Omega _s}\boldsymbol{\alpha }_s:\zeta _y(\dot{\textbf{u}}_s^{(1)})\,\mathrm d\textbf{y} -\int _{\Omega _m}\nabla _y\cdot \textbf{w}_m^{(1)}\,\mathrm d\textbf{y} -\int _{\Omega _s}\nabla _y\cdot \textbf{w}_s^{(1)}\,\mathrm d\textbf{y}. \end{aligned}$$

Applying the divergence theorem to the final two terms, and using periodicity and the interfacial flux-continuity condition, these terms cancel. Introducing

B19 $$\begin{aligned} \textbf{w}_{\textrm{eff}}{:}{=}\langle \textbf{w}_m^{(0)}\rangle _m+ \langle \textbf{w}_s^{(0)}\rangle _s, \end{aligned}$$

we obtain

B20 $$\begin{aligned} \frac{\langle \dot{p}_m^{(0)}\rangle _m}{\langle M_m\rangle _m} +\frac{\langle \dot{p}_s^{(0)}\rangle _s}{\langle M_s\rangle _s}&=-(\langle \boldsymbol{\alpha }_m\rangle _m+\langle \boldsymbol{\alpha }_s\rangle _s):\zeta _x(\dot{\textbf{u}}^{(0)}) -\nabla _x\cdot \textbf{w}_{\textrm{eff}} \nonumber \\&\quad -\langle \boldsymbol{\alpha }_m:\zeta _y(\dot{\textbf{u}}_m^{(1)})\rangle _m -\langle \boldsymbol{\alpha }_s:\zeta _y(\dot{\textbf{u}}_s^{(1)})\rangle _s. \end{aligned}$$

Using the displacement correctors (56a)–(56b) and differentiating with respect to time gives

B21 $$\begin{aligned} \frac{\langle \dot{p}_m^{(0)}\rangle _m}{\langle M_m\rangle _m} +\frac{\langle \dot{p}_s^{(0)}\rangle _s}{\langle M_s\rangle _s}&=-\nabla _x\cdot \textbf{w}_{\textrm{eff}} -(\langle \boldsymbol{\alpha }_m\rangle _m+\langle \boldsymbol{\alpha }_s\rangle _s):\zeta _x(\dot{\textbf{u}}^{(0)}) \nonumber \\&\quad -(\langle \mathbb {L}_m^{\mathrm T}:\boldsymbol{\alpha }_m\rangle _m +\langle \mathbb {L}_s^{\mathrm T}:\boldsymbol{\alpha }_s\rangle _s):\zeta _x(\dot{\textbf{u}}^{(0)}) \nonumber \\&\quad -\langle \boldsymbol{\alpha }_m:\boldsymbol{\tau }_m\rangle _m\dot{p}_m^{(0)} -\langle \boldsymbol{\alpha }_s:\boldsymbol{\tau }_s\rangle _s\dot{p}_s^{(0)} \nonumber \\&\quad -\langle \boldsymbol{\alpha }_s:\tilde{\boldsymbol{\tau }}_s\rangle _s\dot{p}_m^{(0)} -\langle \boldsymbol{\alpha }_m:\tilde{\boldsymbol{\tau }}_m\rangle _m\dot{p}_s^{(0)}. \end{aligned}$$

Rearranging (B21) gives (65) in the main text.
